# Tuning the Response of GPCR-Based Yeast Sensors Using Fluorescent Reporters

**DOI:** 10.1021/acssynbio.5c00466

**Published:** 2025-12-15

**Authors:** Ryan Langevin, McKenna Martin-Downey, Amisha Patel, Haden Archer, Sara J. Davila Severiano, Pamela Peralta-Yahya

**Affiliations:** † School of Chemistry and Biochemistry, 1372 Georgia Institute of Technology, Atlanta, Georgia 30332, United States; ‡ School of Chemical & Biomolecular Engineering, 1372 Georgia Institute of Technology, Atlanta, Georgia 30332, United States; § Bioengineering Graduate Program, 1372 Georgia Institute of Technology, Atlanta, Georgia 30332, United States

**Keywords:** human GPCRs, biosensors, fluorescent proteins, yeast

## Abstract

G protein-coupled receptors (GPCRs) recognize ligands on the cell surface, initiating intracellular signaling pathways that control a variety of biological processes, from neurotransmission and hormone regulation to light detection and smell. As entryways into these pathways, GPCRs are key pharmacological targets, with 30% of FDA-approved drugs targeting them. High-throughput GPCR-based sensors in yeast are proven platforms for the identification of novel GPCR ligands. Most human GPCRs (hGPCRs), however, led to small increases in the signal after activation, hindering the development of high-throughput (HT) assays. To streamline the generation of HT assays for biomedically important hGPCRs, here we analyze five fluorescent reporters in the context of hGPCR-based sensors. Using the serotonin receptor 4 (HTR4)-based sensor as a testbed, we identify YPet, a yellow fluorescent protein previously evolved for improved intracellular fluorescence, as the optimal fluorescent reporter when using flow cytometry, fluorescence-activated cell sorting, or a fluorescent plate reader. YPet increases the dynamic range of hGPCR-based sensors in general, enabling the engineering of HTR4-, MC4R- S1PR2-, HTR1A-, and Mel1A-based sensors with vastly higher increases in signal than previously engineered sensors. YPet even allowed the construction of a functional HTR1D-based sensor, a sensor that had been difficult for the field to construct. Finally, the fast maturation of YPet reduces the time to readout from 4 h to 30 min, unlocking point-of-care diagnostic applications previously inaccessible to hGPCR-based sensors in yeast. Looking ahead, the identification of YPet as the optimal fluorescent reporter for yeast hGPCR-based sensors opens the door to the standardized generation of hGPCR high-throughput assays in this host, and sets the stage for ultrahigh-throughput single-cell experiments toward the identification of new ligands for known GPCRs, GPCR deorphanization, and GPCR engineering to bind designer ligands.

## Graphical abstract

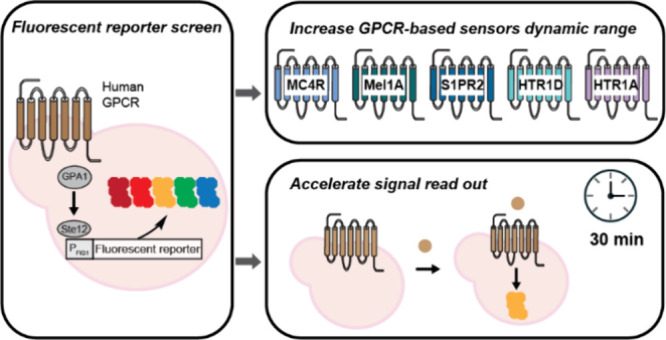

## Introduction

G protein-coupled receptors (GPCRs) detect stimuli on the cell surface, working as knobs to control different biological functions, from neurotransmission and energy metabolism to immune response. The centrality of GPCRs in cell signaling implicates them in multiple conditions, including depression,^1^ diabetes and obesity,^2^ glaucoma,^3^ neurodegenerative diseases,^4^ and cancer.^5^ Today, more than 30% of FDA-approved drugs target GPCRs,^6^ with more than 500 drug candidates in clinical trials.^7^

High-throughput assays in rapidly growing organisms, such as the yeast *Saccharomyces cerevisiae*, are valuable tools for GPCR ligand discovery.^8^ In yeast GPCR-based sensors, a human GPCR (hGPCR) is expressed on the cell surface. Ligand activation of the hGPCR leads to coupling to the yeast mating pathway, resulting in reporter gene transcription leading to cell growth, fluorescence, or luminescence.^9^ Yeast’s robustness and rapid doubling time allow it to be used as a first-line high-throughput screening platform to identify hGPCR activating ligands, which can be validated via secondary assays in mammalian cells and, ultimately, in animals.

A key challenge with hGPCR-based sensors in yeast is that most hGPCRs result in modest increases in signal after ligand activation (Table 1). Reasons for this outcome include poor functional expression of hGPCR on the yeast cell surface and/or poor coupling of the hGPCR to the yeast machinery.^10^ Successfully implemented strategies toward improving the functional expression of hGPCRs in yeast include expression of the hGPCR from different strength promoters in plasmids with increased copy number,^10,11^ appendage of secretion tags to the GPCR to improve their transit to the cell surface,^12^ and changes in yeast membrane composition to better mimic the human cell membrane.^13^ Toward improving hGPCR coupling to the yeast machinery, a successful strategy has been the use of yeast G_α_/human G_α_ chimeras, with the human G_α_ portion interacting with the hGPCR and the yeast G_α_ portion interacting with the yeast machinery.^14–17^ More recently, the use of hGPCR isoforms has also been shown to successfully improve sensor signal.^18^ Finally, strategies that focus on the mechanism of the reporter gene have also been fruitful for increasing the overall sensor signal. For example, transitioning from fluorescent reporters that depend on protein accumulation to enzymatic-based reporters, such as luminescence, and using synthetic transcription factors at the end of the scaffolded signaling cascade can amplify overall sensor output.^11,19–21^

**Table 1:** Fluorescence Reporters Used in the Context of human GPCR-Based Yeast Sensors^a^

reporter	human GPCR	GPCR promoter	G_α_	ligand	signal after activation^b^	chemical exposure time^c^	ref
eGFP	OR1G1	P_TEF1_	GPA1	decanoic acid	30	4 h	^11^
	OR2A7			lilial	∼3		
	OR2T4			pinene	3		
	OR10S1			lilial	∼4		
	HTR4B			serotonin	3		^22^
					13		this work
ENVY GFP	μ opioid receptor	P_CCW12_	GPA1/G_i3_	[d-Ala^2^, *N*-MePhe^4^, Gly-ol]-enkephalin	44.3	8 h	^13^
ZsGreen	CB1R	P_CCW12_	GPA1/G_αi/o_	arachidonyl-2′-chloroethylamide	93.6	8 h	^29^
	CB2R	P_CCW12_	GPA1/G_αi1_	CP55940	∼19	4.6 h	^23^
sfGFP	HTR4B	P_CCW12_	G_αz_	serotonin	64	4 h	^17^
	A2BR	P_CCW12_	GPA1	adenosine	∼325		^21^
	Mel1A	P_HHF2_	GPA1	melatonin	∼150		
	FFA2R	P_CCW12_	GPA1/Gα_i1_	acetate	∼11	3 h	^25^
mTq2	HTR4B	P_TEF1_	GPA1–5aaG_15_	serotonin	∼12	18 h	^16^
	S1PR1		GPA1–5_aa_G_T_	sphingosine-1-phosphate	∼7		
	S1PR2		GPA1–5_aa_G_12_		∼8		
	S1PR3		GPA1–5_aa_G_*z*_		∼6		
	SSTR5		GPA1–5_aa_G_i_	SRIF-14	∼7		
	Mel1A		GPA1–5_aa_G_i_	melatonin	∼10		
	Mel1B		GPA1–5aaG_Z_		∼6		
	ADORA2_A_		GPA1–5_aa_G_15_	adenosine	∼6		
	ADORA2_B_		GPA1–5_aa_G_Z_		∼12		
	ADORA1		GPA1–5_aa_G_O_		∼9		
	PTGER3		GPA1–5_aa_G_T_	prost. E2	∼8		
	CNR2		GPA1–5_aa_G_Q_	HU-210	∼3		
	AVPR2		GPA1–5_aa_G_S_	[Arg^8^]-vasopressin	∼10		
	SUCNR1		GPA1–5_aa_G_*z*_	succinate	∼8		
	PTAFR		GPA1–5_aa_G_i_	PAF	∼6		
	CHRM1		GPA1–5_aa_G_13_	acetylcholine	∼5		
	CHRM3		GPA1–5_aa_G_*z*_		∼5		
	FFAR2		GPA1–5_aa_G_*z*_	acetate	∼3		
	LPAR1		GPA1–5_aa_G_13_	lysophosphatidic acid	∼4		
	GPR35		GPA1–5_aa_G_13_	kynurenic acid	∼2		
	PTGER3		GPA1–5_aa_G_T_	prost. E2	∼9		
	MRGPRD		GPA1–5_aa_G_S_	β-alanine	∼1		
	ADRA2_A_		GPA1–5_aa_G_Z_	epinephrine	∼12		
	ADRA2_B_		GPA1–5_aa_G_T_	epinephrine	∼12		
	HCAR2		GPA1–5_aa_G_15_	niacin	∼6		
	HCAR3		GPA1–5_aa_G_i_	3-hydroxyoctanoate	∼5		
	SSTR5		GPA1-Gα_Z_	SRIF-14	∼40		^37^
mCherry	P2Y2	P_TDH3_	GPA1/G_iα3_	ATP	∼15	6 h	^24^
mScarlet	HTR4B	P_TEF1_	GPA1	serotonin	14	4 h	this work
mKate2	HTR4B	P_TEF1_	GPA1	serotonin	41	4 h	this work
YPet	MC4R	P_TEF1_	GPA1	bremelanotide	221	4 h	this work
	S1PR2	P_TEF1_		CYM-5520	179		
	Mel1A	P_TEF1_		melatonin	173		
	HTR4B	P_ADH1_		serotonin	89		
	HTR1D	P_TEF1_			27		
	HTR1A	P_TEF1_/P_ADH1_			27		

a Sensors in this table use the Ste12/mating pathway promoter to drive reporter gene transcription. For sensors with alternative transcription factor/promoter pairs, see Supporting Table 1 **.**

b Reported as fold increase signal after activation in the presence of the indicated ligand.

c Time that the sensor was incubated in the presence of the ligand.

An underexplored strategy to increase the signal of hGPCR-based sensors is the fluorescent reporter choice. To date, only green (GFP), cyan (mTq2), and red (mCherry) fluorescent proteins have been used as reporters of hGPCR-based sensors.^11,13,16,17,22–28^ As shown in Table 1 (an extended version in Table SI1), most fluorescence hGPCR-based sensors have reached an average 10-fold increase in signal after activation. There are, however, some remarkable outliers, such as the A2DR (∼325-fold)-, Mel1A (∼150-fold)-, and HTR4-based sensors (64-fold), which relied on a highly engineered yeast strain (13 deletions) and the use of a synthetic transcription factor to focus GPCR signal to fluorescence reporter expression.^17,21^ Another exception is the CB1R-based sensor (∼94-fold), where improved signal is obtained by using a syn-prepro secretion sequence and a truncated version of C1BR.^29,18^ Even with this rather limited dynamic range, fluorescence-based hGPCR sensors in yeast have enabled (1) the detection of serotonin,^30^ melatonin,^31^ and tetrahydrocannabinols^29^ produced by engineered yeast, (2) the differential detection of microbially produced medium-chain fatty acids by engineered bacteria,^32^ and (3) the screening of >300 human metabolites against 30 unique hGPCRs, each coupled to one of 10 possible G_α_ subunits to identify ligands for orphan hGPCRs, with the majority of interactions being validated in mammalian cells.^16^

Improving the dynamic range of fluorescence hGPCR-based sensors is critical for increasing the resolution of single-cell experiments, lowering the false-negative rate of high-throughput screens and enabling the identification of ligands previously overlooked in the baseline of the screen. It will also allow access to ultrahigh-throughput single-cell experiments toward (1) the evolution of GPCRs to bind user-specific ligands (Designer Receptors Exclusively Activated by Designer Drugs^33^) that can be used as chemogenetic tools to understand cell signaling, (2) GPCR deorphanization, and (3) the identification of new GPCR targets for known ligands toward reusing known drugs for other diseases. As fluorescent protein properties vary *in vitro* and *in vivo* and depend on the intracellular microenvironment, systematic studies have measured the brightness,^34,35^ p*K*_a_,^35^ and maturation^36^ of subsets of fluorescent proteins in yeast. Although valuable, these studies report fluorescent protein properties in relation to the proteins in the study, using different yeast strains (S288C, CEN-PK), optical techniques (microscopy, flow cytometry), conditions (cell density, media), and gene expression strategies (promoters, T2A expression).

In this work, we evaluated five fluorescent proteins with different spectral and biophysical properties as reporters for hGPCR-based sensors, with the goal of identifying the fluorescent reporter with the highest increase in signal after activation. Using a serotonin receptor 4 (HTR4)-based sensor as a testbed, we compared the increase in signal after activation in the presence of serotonin using YPet, mKate2, mScarlet, mTq2, and eGFP as reporters. YPet outperformed all other fluorescent reporters, achieving a 57-fold increase in signal in a stably integrated strain. The ability of YPet to increase the signal after activation of hGPCR-based sensors is general, enabling the construction of sensors for four biomedically important hGPCRs: melatonin receptor 1A (Mel1A), melanocortin 4 receptor (MC4R), sphingosine-1-phosphate receptor 2 (S1PR2), and serotonin receptor 1A (HTR1A). The dynamic range of the engineered Mel1A-, MC4R-, S1PR2-, and HTR1A-based sensors surpassed that of the previously engineered sensors. Furthermore, it allowed the construction of a functional serotonin receptor 1D (HTR1D)-based sensor, which has been a challenge for the field. Finally, the fast maturation of YPet enabled time compression from chemical detection to read out from 4 hours to 30 min, opening the door to point-of-care diagnostic applications. Looking ahead, the identification of YPet as the optimal fluorescence reporter for hGPCR-based sensors in yeast sets the stage for ultrahigh-throughput single-cell experiments toward the identification of new ligands for known GPCRs, GPCR deorphanization, and GPCR engineering to bind designer ligands.

## Results

### Fluorescent Protein Reporter Properties

In this work, we evaluate the performance of five fluorescent proteins in the context of hGPCR-based sensors (Figure 1A). Given that *S. cerevisiae* has strong autofluorescence due to the presence of tryptophan, pyridoxine, and riboflavin,^38^ the choice of fluorescent reporter is critical as the fluorescent protein spectrum could overlap with that of *S. cerevisiae*, potentially reducing the increase in signal after activation. Specifically we evaluated (1) eGFP, a green fluorescent protein with enhanced stability and brightness as compared to GFP,^39^ (2) mKate2,^40^ a monomeric far-red fluorescent protein that is fast-maturing, acid-resistant, and photostable, (3) mScarlet,^41^ a monomeric red fluorescent protein with improved brightness when compared to coral red fluorescent proteins, (4) YPet,^42^ a yellow fluorescent protein evolved for improved intracellular fluorescence, and (5) mTq2, a cyan fluorescent protein with increased brightness and photosensitivity in acidic conditions.^43^ As shown in Figure 1B, the excitation and emission spectra of eGFP, mTq2, mScarlet, and YPet overlap with that of riboflavin (a large component of yeast autofluorescence), while mKate2 is red-shifted and shows minimum overlap with riboflavin.^44^ Brightness, which takes into account quantum yield and extinction coefficient, is highest for YPet, while it is lowest for mKate2 (Figure 1C). The p*K*_a_ of the fluorescent protein is also important as the yeast media in hGPCR-based sensors is set to pH = 7 to mimic its behavior in mammalian systems. Except for mTq2 (p*K*_a_ = 3.1), all fluorescent proteins have a p*K*_a_ between 5.3 and 6.. Given differences in spectral overlap with riboflavin, protein brightness, and p*K*_a_, it is not obvious *a priori* which fluorescent protein may be the best reporter in hGPCR-based sensors.

**Figure 1:**
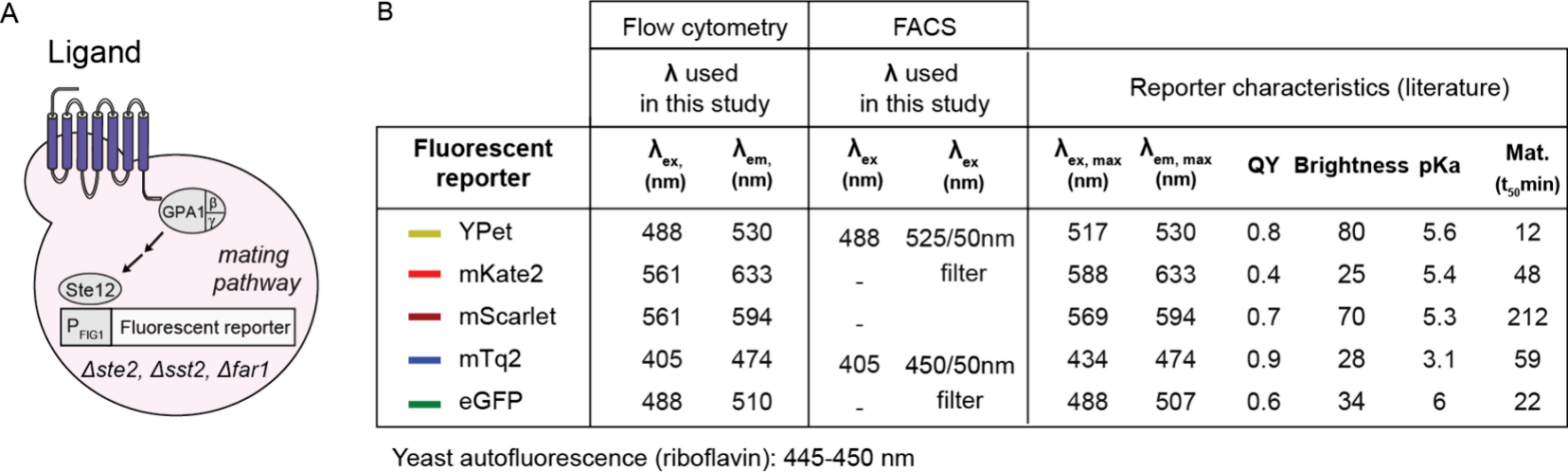
Fluorescent protein reporters used in the context of yeast human GPCR-based sensors. (A) Schematic of a heterologous hGPCR (blue) coupling to the yeast mating pathway (gray), ultimately resulting in the expression of a fluorescent reporter. (B) Table of fluorescent reporter properties. Shown are the emission and excitation wavelengths used for flow cytometry and fluorescence-activated cell sorting (FACS) measurements in this study. Also shown are maximum excitation wavelength, maximum emission wavelength, quantum yield, brightness, and p*K*_a_ values obtained from FPbase^44^ of the five fluorescent proteins in this study, and maturation time at 32 °C obtained from Balleza et al.^45^

### Evaluation of Fluorescent Reporters Using the HTR4-Based Sensor as a Testbed

To compare the performance of YPet, mKate2, mScarlet, mTq2, and eGFP, we used the HTR4-based sensor that couples to the yeast mating pathway via the yeast G_α_ subunit GPA1.^22^ We expressed HTR4 from the strong P_TEF1_ promoter either in a multicopy plasmid (Figure 2A), single integrated at the *His3* locus of the GPCR sensor strain (PPY140: W303 Δ*ste2*, Δ*far1*, Δ*sst2* ^11^) (Figure 2B), or double integrated at both the *His3* and *Trp1* loci of PPY140 (Figure 2C). In all cases, the fluorescent reporters were under control of the mating pathway promoter Fig1 (P_Fig1_) in a centromeric single-copy plasmid. For all sensor experiments, the chemical incubation time was kept constant at 4 h to allow fluorescent proteins to mature. To describe the dynamic range of the sensors, we quantified the fold increase in signal after activation, i.e., signal in the presence of serotonin divided by the signal in the presence of the carrier solvent, DMSO. The operational range of the sensor is defined as the signal between logEC_10_ and logEC_90_.

**Figure 2:**
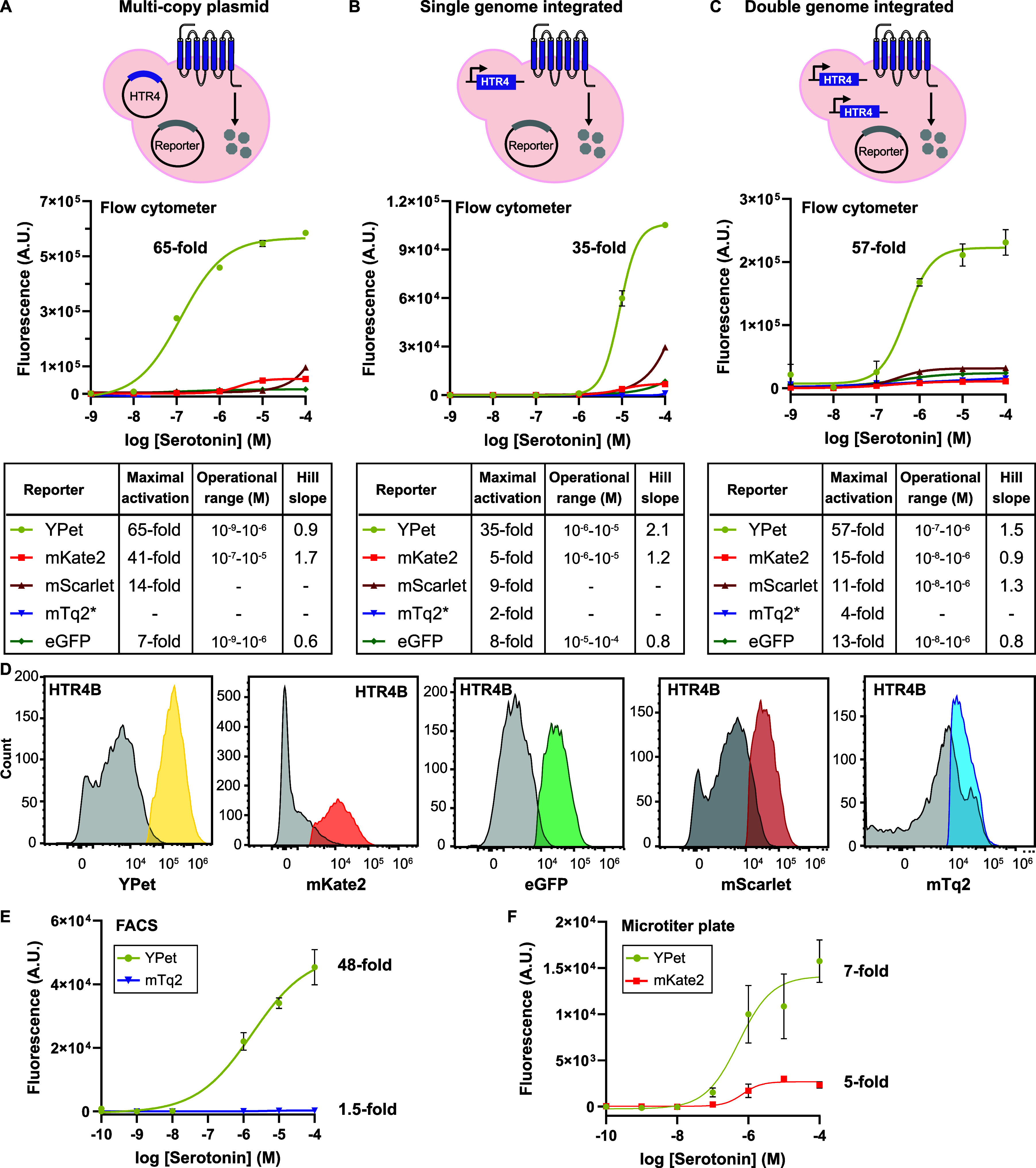
Performance of yeast HTR4-based sensors using five fluorescent reporters. Dose–response curves of the (A) multicopy plasmid version, (B) single-genome integrated version, and (C) double-integrated version of the HTR4-based sensor in the presence of serotonin using YPet, mKate2, mScarlet, GFP, or mTq2* (mTq2:D134E) as the reporters. The fluorescence data were acquired after a 4 h incubation with serotonin. Data were fitted using a nonlinear regression variable slope (four parameter) using GraphPad. For (A), shown are mean ± standard error of the mean (SEM) of three technical replicates, *n* = 3. For (B) and (C) shown are mean ± SEM of three biological replicates, *n* = 3. The tables display the maximal activation as a fold increase in signal in the presence of serotonin divided by the signal in the presence of the carrier solvent (DMSO), operational range (signal between logEC_10_ and logEC_90_), and Hill slope. -: Unstable nonlinear regression curve. Individual curves of each of the fluorescent proteins can be found in Figure S2. (D) Sample histograms of the double-integrated HTR4-based sensor with YPet, mKate2, eGFP, mScarlet, and mTq2 as the reporters. Gray: signal of the sensor in the presence of the carrier solved DMSO. Colors represent the activated populations of different fluorescent reporters. (E) Dose–response of the double-integrated HTR4-based sensor with YPet or mTq2 wild type as the reporter using a fluorescent-activated cell sorter (Sony FACS SH800) for signal readout. (F) Dose–response of the double-integrated HTR4-based sensor with YPet or mKate2 as the reporter using a fluorescent plate reader (Tecan Infinite M1000 Pro) for signal readout. For (E) and (F), shown are mean ± SEM of three biological replicates, *n* = 3. Data were fitted using a nonlinear regression variable slope (four parameter).

For all versions of the HTR4-based sensor, i.e., multicopy plasmid, single integrated, and double integrated, using YPet as the fluorescent reporter delivered the largest increase in signal after activation (Figure 2). For the multicopy plasmid version of the sensor, we first performed an on–off screen of three independent colonies due to large colony-to-colony variation to identify the strain leading to the highest increase in signal after activation in the presence of serotonin (Figure S1). Using the best performing strain for each fluorescent reporter, we performed a dose–response curve in technical replicates (Figures 2A and S2). Using YPet as the reporter, the multicopy plasmid version of the HTR4-based sensor achieved a 65-fold increase in signal after activation in the presence of 10^–4^ M of serotonin, with an operational range between 10^–9^–10^–6^ M serotonin. Using mKate2 as the reporter, a 41-fold increase in signal is achieved, while mScarlet and eGFP led to 14-fold and 7-fold increases, respectively.

Next, we evaluated the single-integrated HTR4-based sensors in biological triplicate (Figures 2B and S2). YPet outperformed all other reporters, achieving a 35-fold increase in signal after activation in the presence of 10^–4^ M serotonin with an operational range between 10^–6^ and 10^–5^ M serotonin. Noteworthy, all fluorescent proteins drop in dynamic range when compared to their multicopy plasmid-based versions due to the fewer number of HTR4 copies in the cell.

Then, we assessed the double-integrated HTR4-based sensor with all five fluorescent proteins (Figures 2C and S2). YPet again outperformed all other reporters, reaching a 57-fold increase in signal after activation in the presence of 10^–4^ M serotonin with an operational range between 10^–7^–10^–6^ M serotonin. The second-best fluorescent reporter was mKate2, achieving a 15-fold increase in signal after activation with an operational range from 10^–8^–10^–6^ M serotonin. The fluorescent proteins mScarlet and eGFP also performed well in the double-integrated versions, achieving 11-fold and 13-fold increases in signal activation, respectively. The superior performance of YPet can be clearly observed in the flow cytometry histograms (Figure 2D).

Underwhelmed by the underperformance of mTq2, which in one study has been used to successfully construct 21 sensors using an 18-h chemical incubation time,^16^ we went back to its DNA sequence and found a point mutation, mTq2:D134E. After reverting the point mutation, we reanalyzed the performance of mTq2 wild type in the double genome-integrated version of the HTR4-based sensor using our standard 4-h chemical incubation time, but no improvement in signal after activation was observed (Figure S3).

For all flow cytometry experiments, we used the Cytek Aurora 5L. It is possible that the Cytek Aurora 5L excitation and emission wavelengths (405/474 nm) may not have been optimal for the detection of mTq2. Thus, we measured the signal after activation of the HTR4-based sensor using mTq2 or YPet as the reporter, using a Sony SH8000 fluorescent-activated cell sorter (FACS). As shown in Figure 2E, using the 405 nm excitation laser and a 450/50 nm filter to measure mTq2 emission, the HTR4-based sensor using mTq2 as the reporter resulted in a 1.5-fold increase in signal after activation. Using the 488 nm laser and a 525/50 nm filter to measure YPet emission, the HTR4-based sensor using YPet as the reporter resulted in a 48-fold increase in signal after activation.

Finally, as flow cytometry or FACS may not be readily available in synthetic biology laboratories, we tested the HTR4-based sensor using all five fluorescent reporters in a Tecan Infinite M1000 Pro fluorescent plate reader. As shown in Figure 2F, only YPet and mKate2 resulted in a significant increase in signal after activation, with YPet achieving a 7-fold increase in signal after activation and mKate achieving a 5-fold increase. Interestingly, mTq2 did not result in a signal over background (Figure S4).

Taken together, in the context of hGPCR-based sensors, YPet leads to a much larger dynamic range independent of whether the hGPCR is expressed from a multicopy plasmid, single integrated, or double integrated in the yeast genome. Furthermore, YPet outperformed the other four fluorescent proteins not only when using flow cytometry, but also when using FACS or a fluorescent plate reader. We hypothesize that the slower maturation of mTq2 over YPet and mKate2 (Figure 1C) affects the performance of mTq2 in a rapid 4-h chemical incubation experiment. Taken together, YPet is the optimal fluorescent reporter in the context of hGPCR-based sensors when using a short 4-h chemical incubation time.

### Tuning the Sensor Output by Varying the Promoter Strength and GPCR Integration Locus

The effect of promoter strength has been well-investigated in the context of the native yeast GPCR Ste2, where an increase in promoter strength leads to an increase in GPCR sensor response.^21^ However, optimizations performed using native Ste2 do not necessarily hold true when using heterologous human GPCRs. For example, increasing the number of Ste2 copies in the genome does not result in a major increase in signal after activation,^21^ while increasing the number of heterologous expressed hGPCRs does increase the signal after activation.^18,37^ This is likely due to the yeast endogenous regulation of Ste2. To our knowledge, mostly strong promoters, including P_TEF1_,^11,37^ P_CCW12_,^13,17,21^ and P_TDH3_,^24^ have been used to drive expression of hGPCRs.

We investigated the use of the medium-strength promoter P_ADH1_ in the single- and double-integrated versions of the HTR4-based sensor. As Figure 3A shows, the single-integrated P_ADH1_-HTR4-derived sensor results in no increase in signal after activation, while the single-integrated P_TEF1_-HTR4-derived sensor results in a 35-fold increase. However, the double-integrated P_ADH1_-HTR4-derived sensor reaches an 89-fold increase in thesignal after activation, while the double-integrated P_TEF1_-HTR4-derived sensor reaches a 57-fold increase (Figure 3B). Thus, a strong promoter is favored when generating a single-integration version of a hGPCR-based sensor, while using a medium-strength promoter is preferred when generating a double-integration version. Finally, we explored the contribution of hGPCR integration locus on sensor performance. As Figure 3C shows, the single-integrated P_TEF1_-HTR4-derived sensor at the *Trp1* locus reaches a 44-fold increase in signal after activation, higher than the signal achieved when P_TEF1_-HTR4 is integrated at the *His3* locus (35-fold increase). Integration at the *Trp1* locus leads to a more sensitive sensor, detecting 10^–7^–10^–6^ M serotonin. Consequently, both promoter strength and integration location modulate the dynamic and linear ranges of the hGPCR-based sensors.

**Figure 3:**
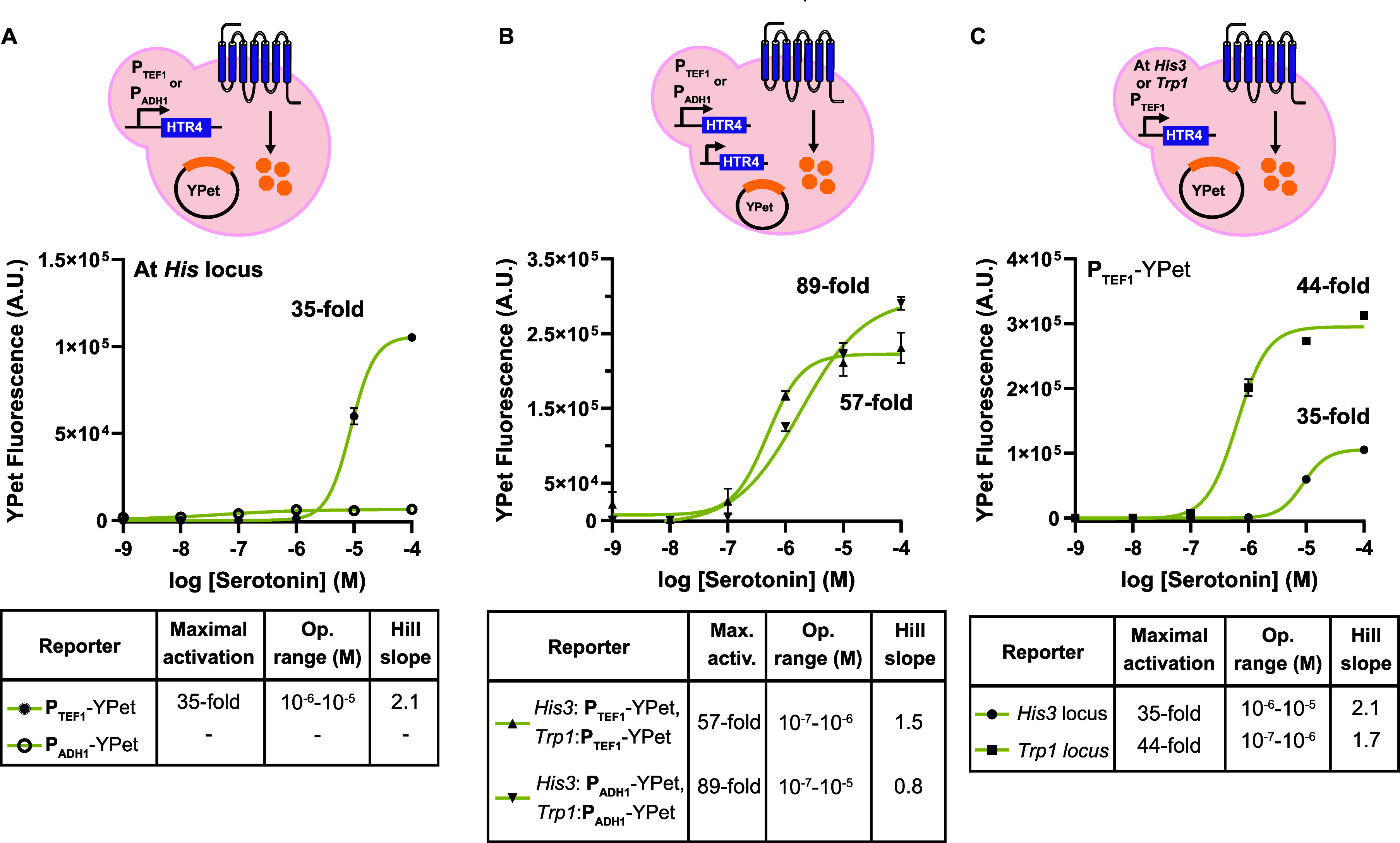
Tuning hGPCR-based sensor output by varying the promoter strength and genome integration location of hGPCRs. (A) Dose–response curve of a single-integrated HTR4-based sensor using the medium-strength promoter P_ADH1_ and the strong promoter P_TEF1_ and YPet as the reporter. (B) Dose–response curve of a double-integrated HTR4-based sensor using the P_ADH1_ or P_TEF1_ and YPet as the reporter. (C) Dose–response curves of a single-integrated P_TEF1_-HTR4-derived sensor at the *His3* or *Trp1* locus of the GPCR sensor strain. For (A–C), fluorescence data were acquired after a 4 h incubation with serotonin, shown are mean ± standard error of the mean of three biological replicates, *n* = 3. The tables display the maximal activation as a fold increase in signal in the presence of serotonin divided by the signal in the presence of the carrier solvent (DMSO), operational range (signal between logEC_10_ and logEC_90_), and Hill slope. -: Unstable nonlinear regression fit.

### Generality of YPet to Increase the Signal after Activation of hGPCR-Based Sensors

Using YPet as the reporter, we constructed sensors for five biomedically important human GPCRs: the G_αs_-coupled melanocortin 4 receptor (MC4R), sphingosine-1-phosphate receptor 2 (S1PR2), and melatonin receptor 1A (Mel1A), as well as the G_αi_-coupled serotonin receptors 1A (HTR1A) and 1D (HTR1D). MC4R is involved in metabolic and body weight regulation.^46^ S1PR2 participates in the regulation of the vascular system including vascular permeability and immune responses.^47^ Mel1A is concerned with the regulation of circadian and circannual rhythms.^44^ HTR1A has been implicated in several physiological functions, including sleep, pain, temperature homeostasis, processing of emotions, and response to stress.^48^ HTR1D is involved in neuromodulation including the central nervous system and physiological processes.^49^

Yeast sensors for MC4R,^50^ S1PR2,^16^ HTR1A,^17,51,52^ and Mel1A^16^ have been previously developed. The MC4R-based yeast sensor relied on a growth-based reporter and achieved a ∼6-fold increase when coupling to the yeast machinery via a GPA1/G_αs_ chimera.^50^ The S1PR2-based sensor using mTq2 as the reporter to achieve an ∼8-fold increase in signal after activation by coupling to the yeast mating pathway via a GPA1/G_α12_ chimera.^16^ The Mel1A-based sensor, using superfolder GFP (sfGFP) as the reporter, achieved a ∼150-fold increase in signal after activation by coupling via GPA1 to the yeast machinery.^21^ Finally, the most recent HTR1A-based yeast sensor relied on a GPA1/G_αz_ chimera to couple to the yeast machinery and used superfolder GFP as the reporter to achieve a 1.5-fold increase in signal after activation.^17^

We hypothesized that YPet would result in MC4R-, S1PR2-, HTR1A-, and Mel1A-based sensors with improved dynamic range. We constructed the multicopy plasmid, single-integrated, and double-integrated version of these sensors. We used P_TEF1_ to drive MC4R, S1PR2, and Mel1A expression. In the case of the HTR1A-based sensor, we used P_TEF1_-HTR1A at the *Trp1* locus and P_ADH1_-HTR1A at the *His3* locus. As shown in Figure 4A, the double genome-integrated version of the MC4R-based sensor achieved a 102-fold increase in signal after activation with 10^–4^ M bremelanotide, a known MC4R agonist. This signal is 40 times higher than that of the previous MC4R-based sensor. Figure 4B shows that the double-integrated version of the S1PR2-based sensor achieved a 181-fold increase in signal after activation with 10^–5^ M CYM-5520, a selective allosteric agonist of S1PR2,^53^ with an operational range of 10^–8^ to 10^–6^ M CYM-5520. This increase in signal after activation is 35-fold higher than the previously reported S1PR2-based sensor. Of note, previous work used sphingosine-1-phosphate (S1P) as the agonist rather than CYM-5520, which in mammalian cells result in similar activation levels.^53^ Finally, the double-integrated version of the HTR1A-based sensor achieved a 27-fold increase in signal after activation with 10^–3^ M serotonin (Figure 4C), 18 times higher than that of the previously reported sensor.

**Figure 4:**
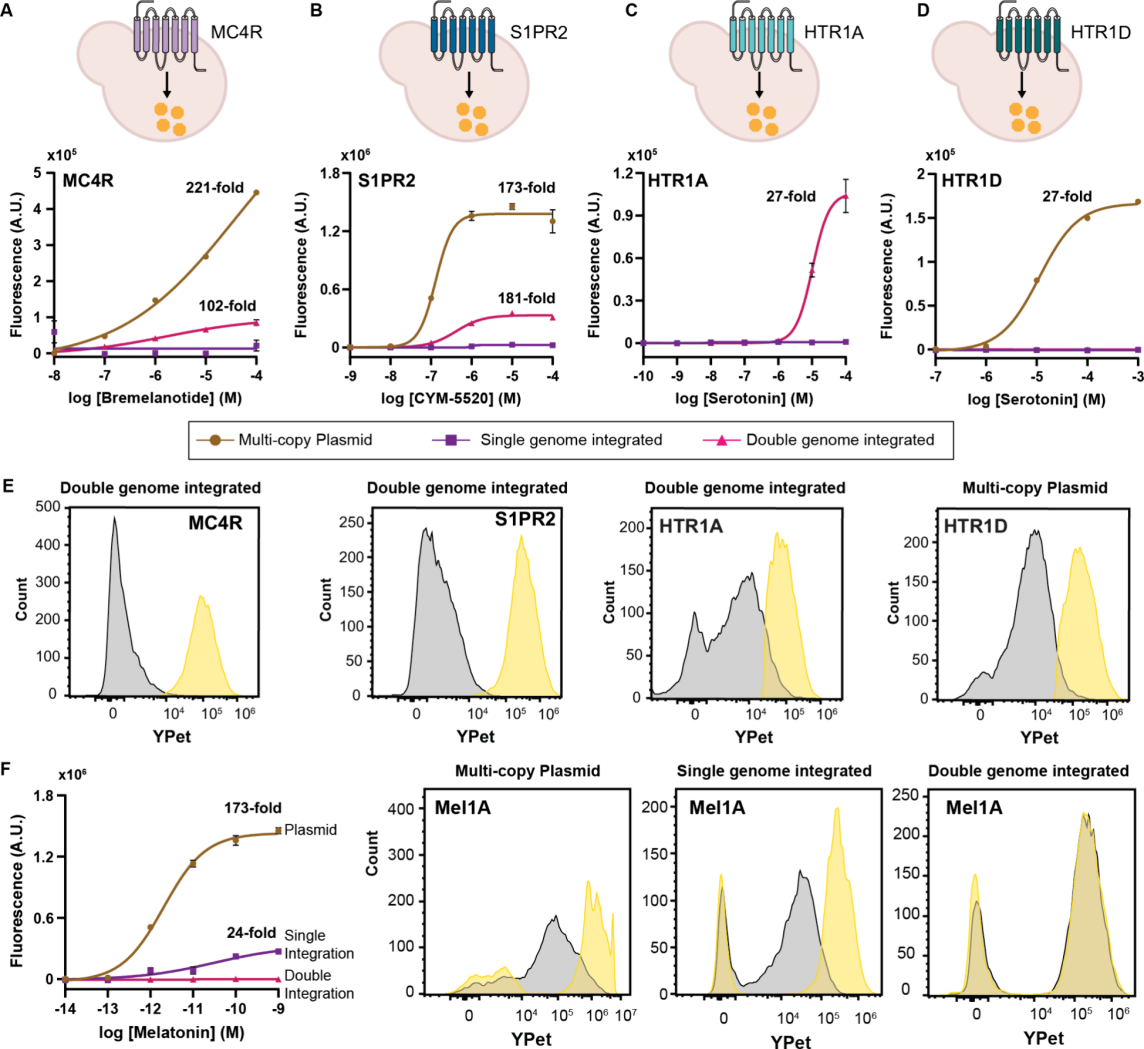
Generality of YPet as a fluorescent reporter for hGPCR-based sensors. (A–D) Dose–response curves of the multi-copy plasmid, single-integration, and double-integration version of the MC4R-, S1PR2-, HTR1A-, and HTR1D-based sensors. The data for the plasmid version of the sensors were obtained in technical triplicate. The data for the integrated versions of the sensors were obtained in biological triplicate. Shown is the mean ± standard error of the mean (SEM) of technical (in the case of the plasmid-based sensor) or biological (in the case of the integrated-based sensor) triplicates, *n* = 3. Data were fitted using a nonlinear regression variable slope (four parameters) using GraphPad. (E) Sample histograms of double-integrated versions of the MC4R-, S1PR2-, and HTR1A-based sensors and the multi-copy plasmid version of the HTR1D-based sensors using YPet as the reporter. Gray: signal of the sensor in the presence of the carrier-solved DMSO. Yellow: YPet signal of activated population. (F) Dose–response curves of the multi-copy plasmid, single-integration, and double-integration versions of the Mel1A-based sensor, and sample histograms for the activated population. In the double-integration version of the Mel1A-based sensor, no activation was observed. Shown is the mean ± SEM of three technical (in the case of the plasmid-based sensor) or biological (in the case of the integrated-based sensor) triplicates, *n* = 3. Data were fitted via a nonlinear regression variable slope (four parameters).

Unlike other hGPCR-based sensors, all versions of the Mel1A-based sensor showed high basal activation, as seen by the bimodal distribution of the population’s fluorescence in the off-state, i.e., in the presence of the carrier solvent DMSO (Figure 4F). In the multicopy plasmid version of the sensor, the bimodal distribution is not very accentuated, and the addition of melatonin shifts most of the population to a highly active state, achieving a 173-fold increase in signal after activation. However, in the single- and double-integrated versions of the Mel1A-based sensor, there is a well-defined bimodal distribution in the off-state. In the single-integrated version of the sensor, addition of melatonin shifts the population, achieving a 24-fold increase in signal after activation. However, there is no shift observed in the double-integrated version of the sensor upon melatonin addition. Thus, it may be more difficult to construct sensors that show a bimodal distribution in the off-state. Hypothesizing that a change in promoter may alter the population’s fluorescence distribution, we generated a P_ADH1_-Mel1A-derived double-integrated sensor, but it also proved to be nonfunctional (Figure S4).

The field has struggled to make an HTR1D sensor, and we hypothesize that YPet could result in the generation of a functional sensor. In 2000, Brown et al. reported an HTR1D-based sensor that coupled via GPA1–5aaG_αi3_ to the yeast machinery and used LacZ as the reporter gene.^51^ Although an EC_50_ was reported (135 nM), no fold increase in signal after activation was disclosed. In 2022, a similar HTR1D sensor was attempted using GPA1–5aaG_αi3_ for coupling and sfGFP as a reporter, but it achieved no increase in signal after activation.^17^ Coupling HTR1D via GPA1 to the yeast machinery also resulted in a nonfunctional sensor.^22^ Generation of a multicopy plasmid version of the HTR1D-based sensor using YPet as the reporter achieved a 27-fold increase in signal after activation in the presence of 10^–3^ M serotonin. Neither the single- nor double-integrated version of the HTR1D-based sensor resulted in an increase in the signal after activation with serotonin. Given that double integration of P_ADH1_- HTR4 resulted in a sensor with higher signal after activation than the double interation of P_TEF1_- HTR4 (Figure 4D), we generated a double-interated P_ADH1_- HTR1D-derived sensor, but we did not observe any sensor functionality (Figure S4).

### Time Dependency on Sensor Fluorescent Reporter Output

The fast maturation time of the fluorescent reporters in this study (Figure 1C) led us to investigate the time dependency of the sensor output. As shown in Table 1, the average chemical incubation time for yeast hGPCR-based sensors in the field is 9 h. In this work, all sensor output has been measured after a 4-h chemical incubation time, thus far. A faster readout for hGPCR-based sensors would not only accelerate the high-throughput process but also open the door to other applications for hGPCR-based sensors, including point-of-care-biomarker detection. Specifically, we measured the fluorescence of the HTR4-based sensor after 0.5-, 1-, 2-, and 3-h chemical incubation times using YPet and mKate2 as the reporters (Figure 5). After just 30 min, YPet resulted in a 13-fold increase in signal after activation in the presence of 10^–4^ M serotonin and had an operational range of 10^–7^–10^–5^ M (Figure 5A). The fluorescent reporter mKate2 achieved a 12-fold increase after a 2 h chemical incubation (Figure 5B). Take together, YPet shortens the time to sensor output to just 30 min. Thus, YPet makes hGPCR-based sensors compatible with point-of-care-applications that demand a fast readout time.

**Figure 5:**
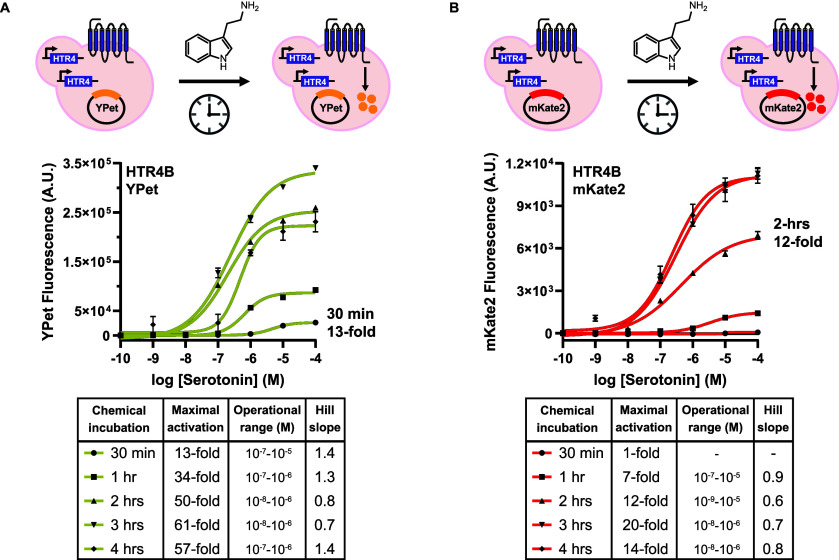
Time dependency of fluorescent reporter output. Fluorescence-based double-integrated HTR4-based sensor as a function of chemical incubation time. (A) YPet was used as the reporter. (B) mKate2 was used as the reporter. Shown are mean ± standard error of the mean (SEM) of three biological replicates, *n* = 3. Data were fitted via a nonlinear regression variable slope (four parameters) using GraphPad. The tables display the maximal activation as fold increase in signal in the presence of serotonin divided by the signal in the presence of the carrier solvent (DMSO), operational range (signal between logEC_10_ and logEC_90_), and Hill slope. -: Unstable nonlinear regression fit.

## Conclusions

In this work, we evaluated the performance of five fluorescent proteins as reporters for hGPCR-based yeast sensors. Using the HTR4-based sensor as a testbed, YPet outperforms mKate2, mScarlet, mTq2, and eGFP in all versions of the sensor tested, i.e., multicopy plasmid, single-integrated, and double-integrated versions. The promoter driving hGPCR expression is important in terms of increasing signal after activation, with the strong P_TEF1_ promoter resulting in a higher increase in signal after activation in the single-integration version of the sensor, whereas the medium-strength P_ADH1_ promoter is a better option when generating a double-integration version. Integration at the *Trp1* locus is also important, with integration at the *Trp1* locus resulting in a higher increase after activation.

The generality of YPet as the optimal fluorescent reporter in the context of hGPCR-based sensors was shown by generating sensors for MC4R, S1PR2, Mel1A, and HTR1A with an increase in signal after activation higher than previous sensors in the literature. Furthermore, using YPet, a plasmid-based version of the HTR1D-based sensor was developed for the first time with a quantifiable signal output.

Point-of-care diagnostics need to be economical, portable, require a small sample quantity, minimally invasive, fast enough to be performed while the patient waits on location, and easy to use. In this work, we have reduced the time to readout of GPCR-based sensors from 4 h to 30 min using YPet as the reporter. Thus, hGPCR-based sensors using YPet as the reporter could be used in the context of point-of-care diagnostic devices, a space previously inaccessible by these cell-based sensors. Additionally, the fluorescent reporter identity and chemical incubation time can also be used to modulate the dynamic and operational ranges of hGPCR-based sensors.

Looking ahead, the identification of YPet as the optimal fluorescent reporter for most applications in the context of GPCR-based sensors will open the doors to single-cell experiments using this platform to facilitate not only hGPCR ligand discovery applications but also the evolution of hGPCRs themselves to bind user-specified ligands.

## Materials and Methods

### Materials

Serotonin hydrochloride (TCI S0370), bremelanotide (Sigma SML2756), melatonin (Sigma M5250), and CYM-5520 (Sigma 5.31371) were used.

### Construciton of Single-Copy Fluorescence Reporter Vectors

The eGFP fluorescent reporter in a single-copy vector, pRS415-Leu2-P_Fig1_-eGFP (pKM586 ^11^), has been previously reported. The fluorescent proteins mKate2, mScarlet, and YPet were cloned into pKM586 by swapping out eGFP to generate pRS415-Leu2-P_Fig1_-mKate2 (pRP986), pRS415-Leu2-P_Fig1_-mScarlet (pHW41), and pRS415-Leu2-P_Fig1‑_YPet (pHW42), respectively. The fluorescent protein mTq2:D134E was cloned into pRS415-Leu2-P_Fig1_-NanoLuc (pEY15 ^54^) by swapping out NanoLuc to generate pRS415-Leu2-P_Fig1_-mTq2:D134E (pMD53). mTq2:D134E was reverted to the wild-type sequence via site-directed mutagenesis to generate pRS415-Leu2-P_Fig1‑_mTq2 (pMD100).

### Construction of Multi-copy hGPCR Vectors Vectors costruction

HTR4 and HTR1D have been previously cloned into multicopy vectors pESC-HIS3-P_TEF1_-HTR4 (pTMC18 ^22^) and pESC-HIS3-P_TEF1_-HTR1D (pAME142 ^22^), respectively. The GPCRs MC4R, S1PR2, Mel1A, and HTR1A were cloned into pESC-HIS3-P_TEF1_-T_CYC1_ (pKM111)^11^ to generate pESC-HIS3-P_TEF1_-MC4R (pMD4), pESC-HIS3-P_TEF1_-S1PR2 (pMD15), pESC-HIS3-P_TEF1_-Mel1A (pTMC3), and pESC-HIS3-P_TEF1_-HTR1A (pAP12), respectively.

### Construction of hGPCR Integration Vectors -His3 Locus

*His3* locus integration vectors were based on pInt-His3-P_ADH1_-FLAG-5-HTR_4B_ (PM137 ^18^). First, P_ADH1_ was replaced with P_TEF1_ by amplifying *S. cerevisiae* P_TEF1_ from pTMC18 and cloned into PM137 to generate pInt-His3-P_TEF1_-FLAG-HTR4 (pSD28). To generate P_TEF1_-GPCRs, MC4R, S1PR2, Mel1A, and HTR1D were cloned into pSD28 by swapping out FLAG-HTR4 to generate pInt-His3-P_TEF1_-MC4R (pRL42), pInt-His3-P_TEF1_-S1PR2 (pRL43), pInt-His3-P_TEF1_-Mel1A (pRL44), and pInt-His3-P_TEF1_-HTR1D (pRL53), respectively. To generate His3 locus integration vectors with P_ADH1_-Mel1A and P_ADH1_-HTR1D, the respective GPCRs were cloned into PM137 ^18^ by swapping out FLAG-HTR4 to generate pInt-His3-P_ADH1_-Mel1A (pRL76) and pInt-His3-P_ADH1_-HTR1D (pRL78), respectively. To generate pInt-His3-P_ADH1_-HTR1A (pAP130), HTR1A was amplified from pAP12 and cloned into pNH603-*C. glabrata* His3-P_ADH1_-MCP-VP64-*C. albicans* T_ADH1_ (pJZC522 ^55^).

### Construction of hGPCR Integration Vectors: Trp1 Locus

*Trp1* locus integration vectors were based on pNH604-P_TetO(1x)_-Venus (pJZC530 ^56^). First, P_TEF1_-FLAG-HTR4 was amplified from pSD28 and cloned into pJZC530 to generate pInt-Trp1-P_TEF1_-FLAG-HTR4 (pRL51). To generate P_TEF1_-GPCRs, MC4R, S1PR2, Mel1A, and HTR1D were cloned into pRL51 by swapping out FLAG-HTR4 to generate pInt-Trp1-P_TEF1_-MC4R (pRL64), pInt-Trp1-P_TEF1_-S1PR2 (pRL65), pInt-Trp1-P_TEF1_-Mel1A (pRL66), and pInt-Trp1-P_TEF1_-HTR1D (pRL67), respectively. To generate pInt-Trp1-P_TEF1_-HTR1A (pAP277), P_TEF1_-HTR1A was cloned into pInt-Trp1-P_TEF1_-GPA1_5AA_Gs (pPM189 ^18^) by replacing GPA1_5AA_Gs. To generate P_ADH1_-hGPCRs, Mel1A and HTR1D were cloned into pInt-Trp1-P_ADH1‑_FLAG-HTR4 (PM290 ^18^) by swapping out FLAG-HTR4 to generate pInt-Trp1-P_ADH1_-Mel1A (pRL77) and pInt-Trp1-P_ADH1_-HTR1D (pRL79), respectively.

### Multicopy Plasmid hGPCR-Based Sensors

PPY140 (*S. cerevisiae* W303 *leu2-3,112 trp1-1 can1-100 ura3-1 ade2-1 his3-11,15* Δ*far1*, Δ*ste2*, Δ*sst2*)^11^ was cotransformed with pTMC18 and pKM586, pRP986, pHW41, pHW42, or pMD53 to generate multicopy plasmid versions of the HTR4-based sensor with GFP (PPY2484), mKate2 (PPY2475), mScarlet (PPY2476), YPet (PPY2474), or mTq2:D134E (PPY2773) as the reporter. To generate multicopy plasmid versions of the MC4R-, S1PR2-, Mel1A-, and HTR1D-based sensors using YPet as the reporter, PPY140 was cotransformed with pHW42 and either pMD4 (PPY2478), pMD15 (PPY2495), pTMC3 (PPY2488), or pAME142 (PPY2504).

### Single-Genome-Integrated hGPCR-Based Sensors

First GPCR integrations occurred at the *His3* locus, unless otherwise noted. The *His3* integration vectors carrying HTR4 (pSD28), MC4R (pRL42), S1PR2 (pRL43), Mel1A (pRL44), HTR1D (pRL53), and HTR1A (pAP130) were linearized by digesting with *PmeI*, and the linearized DNA was integrated into PPY140. Successful integrations were selected on synthetic complete medium with 2% glucose lacking histidine (SD­(H^–^)) and verified via PCR using primers PB140/PB141. The strains generated were PPY140 *HIS3*: P_TEF1_-HTR4 (PPY2785), PPY140 *HIS3*: P_TEF1_-MC4R (PPY2955), PPY140 *HIS3*: P_TEF1_-S1PR2 (PPY2956), PPY140 *HIS3*: P_TEF1_-Mel1A (PPY2957), PPY140 *HIS3*: P_TEF1_-HTR1D (PPY3331), and PPY140 *HIS3:* P_ADH1_-HTR1A (PPY2997). Transformation of PPY2785 with pKM586, pRP986, pHW41, pHW42, or pMD53 resulted in a single-genome-integrated HTR4-based sensor having eGFP (PPY3532), mKate2 (PPY3529), mScarlet (PPY3530), YPet (PPY3528), or mTq2:D134E (PPY3531) as the reporter. Transformation of pHW42 into PPY2955, PPY2956, PPY2957, PPY3331, and PPY2997 resulted in MC4R (PPY2972)-, S1PR2 (PPY2973)-, Mel1A (PPY2974)-, HTR1D (PPY3370)-, and HTR1A (PPY3371)-based sensors using YPet as the reporter. The single-genome-integrated version of the P_ADH1_-HTR4-derived sensor (PPY3844) was obtained by transforming PPY140 *HIS3:* P_ADH1_-HTR4 (PPY2754 ^18^) with pHW42. The single-integration version of the P_TEF1_-HTR4-derived sensor at the *Trp1* locus was obtained by linearizing P_TEF1_-HTR4 (pRL51) and integrating it into PPY140 to generate PPY140 *TRP1*: P_TEF1_-HTR4 (PPY3841). PPY3841 was then transformed with pHW42 to generate the sensor strain (PPY3842) with YPet as the reporter.

### GPCR-Based Sensor Construction—Double-Genome-Integrated Versions

Second hGPCR integrations occurred at the *Trp1* locus, unless otherwise noted. The *Trp1* integration vectors carrying HTR4 (pRL51), MC4R (pRL64), S1PR2 (pRL65), Mel1A (pRL66), HTR1D (pRL67), and HTR1A (pAP277) were linearized by digesting with *PmeI* and were integrated into PPY2785, PPY2955, PPY2956, PPY2957, PPY3331, and PPY2997, respectively. Successful integrations were selected on SD­(HW^–^) plates and verified via PCR using primers PB140/PB142. The strains generated were PPY140 *HIS3*: P_TEF1_-HTR4, *Trp1*: P_TEF1_-HTR4_,_ (PPY3166), PPY140 *HIS3*: P_TEF1_-MC4R, *Trp1*: P_TEF1_-MC4R (PPY3718), PPY140 *HIS3*: P_TEF1_-S1PR2, *Trp1*: P_TEF1_-S1PR2 (PPY3719), PPY140 *HIS3*: P_TEF1_-Mel1A, *Trp1*: P_TEF1_-Mel1A (PPY3720), PPY140 *HIS3*: P_TEF1_-HTR1D, *Trp1*: P_TEF1_-HTR1D (PPY3721), and PPY140 *HIS3*: P_ADH1_-HTR1A, *Trp1*: P_TEF1_-HTR1A (PPY3552). Transformation of PPY3166 with pKM586, pRP986, pHW41, pHW42, pMD53, or pMD100 resulted in a double-genome-integrated HTR4-based sensor having eGFP (PPY3214), mKate2 (PPY3211), mScarlet (PPY3212), YPet (PPY3210), mTq2:D134E (PPY3213), or mTq2 (PPY3733) as the reporter. Transformation of pHW42 into PPY3718, PPY3719, PPY3720, PPY3721, and PPY3552 resulted in MC4R (PPY3722)-, S1PR2 (PPY3723)-, Mel1A (PPY3724)-, HTR1D (PPY3725)-, and HTR1A (PPY3656)-based sensors using YPet as the reporter. Double-genome-integrated sensors containing P_ADH1_-Mel1A and P_ADH1_-HTR1D were generated by integrating PPY140 with either pRL76 and pRL77 (PPY3771) or pRL78 and pRL79 (PPY3773) and transforming with pHW42 to generate P_ADH1_-Mel1A (PPY3765)- and P_ADH1_-HTR1D (PPY3764)-based sensors using YPet as the reporter. A double-integrated version of P_ADH1_-HTR4 was generated by transforming PPY3534 with pHW42, generating PPY3739.

### Flow Cytometry Control Stains

As the unstained control for the plasmid version of the sensors, PPY140 was transformed with pTMC18, pMD4, pMD15, pTMC3, pAME142, or pAME144 to generate PPY2772 (HTR4), PPY2480 (MC4R), PPY2494 (S1PR2), PPY2487 (Mel1A), PPY2505 (HTR1D), and PPY3833 (HTR1A). The unstained controls for the single-integration version of the sensors were PPY2785 (HTR4), PPY2955 (MC4R), PPY2956 (S1PR2), PPY2957 (Mel1A), PPY3331 (HTR1D), and PPY2997 (HTR1A). The unstained controls for the double-integration version of the hGPCR-based sensors were PPY3166 (HTR4), PPY3718 (MC4R), PPY3719 (S1PR2), PPY3720 (Mel1A), PPY3721 (HTR1D), and PPY3552 (HTR1A).

### Chemical Sensing Assay

Overnight cultures of the multicopy plasmid and single-genome-integrated versions of the hGPCR-based sensors and their respective unstained controls were used to inoculate 50 mL of SD­(HL^–^) or 50 mL of SD­(H^–^), respectively (OD_600_ = 0.06). After 18 h at 15 °C (150 rpm), the cultures were centrifuged (3500 rpm, 10 min) and resuspended in SD­(HL^–^) or SD­(H^–^) media to an OD_600_ = 1. To a white, flat-bottomed 96-well plate, 190 μL of pH 7 SD (HL^–^) or SD (H^–^) media, 8 μL of cells, and 2 μL of serotonin (final concentration 0–10^–4^ M for HTR4 and HTR1A, or 0–10^–3^ M for HTR1D), bremelanotide (final concentration 0–10^–4^ M), CYM-5520 (final concentration 0–10^–4^ M), or melatonin (final concentration 0–10^–8.3^ M) were added. Reactions using multicopy plasmid sensors were set up as technical triplicates, with cells aliquoted into the 96-well plate from the same culture. Reactions using integrated sensors were set up as biological triplicates, with three separate transformants being cultured and aliquoted separately. The plate was covered with a sealing membrane, and the sensors were incubated with their respective ligands for 4 h at 30 °C (250 rpm) before reading the results via flow cytometry. For double-integrated sensors, the same protocol was used, with double-integrated sensor strains being cultured with SD­(HLW^–^) and double-integrated unstained control strains being cultured with SD­(HW^–^). For the single-integrated HTR4 sensor integrated at the *Trp1* locus, the same protocol was used, with the sensor strain being cultured with SD­(LW-) and the single-integrated unstained control strain being cultured with SD­(W-). For assays using the Tecan Infinite M1000 Pro, a similar protocol was used, with the overnight cultures being used to inoculate 5 mL (OD_600_ = 0.6) of SD­(HLW-), and a clear, flat-bottom black 96-well plate was used for the 4 h incubation step. For assays using the Sony SH800 Cell sorter, a scaled-up version of this protocol was used. 1.9 mL of pH 7 SD (HLW^–^) or SD (HW^–^) media, 80 μL of cells, and 20 μL of serotonin (final concentration 0–10^–4^ M) were added to a 14 mL polystyrene round-bottom tube for incubation, and cells were subsequently strained using 40 μm Flowmi cell strainers before reading the results via flow cytometry.

### Fluorescence Measurements

For Flow cytometry measurements, fluorescence from 15,000 cells was measured using either a Cytek Aurora 5L Spectral flow cytometer or a Sony SH800 cell sorter. Unless otherwise noted, all measurements were made using the Cytek Aurora. For measurements collected using the Cytek Aurora, the following excitation and emission wavelengths were used (excitation/emission): eGFP (488 nm/510 nm), mKate2 (561 nm/633 nm), mScarlet (561 nm/594 nm), YPet (488 nm/530 nm), and mTq2 (405 nm/474 nm). These numbers can also be found in Figure 1C. After data collection on the Cytek Aurora, spectral unmixing with autofluorescence extraction was performed on SpectroFlo by extracting the background fluorescence of the unstained control population from the fluorescent signals. For measurements collected using the Sony SH800, YPet was excited using a 488 nm laser and was measured using a 525/50 nm filter, and mTq2 was excited using a 405 nm laser and was measured using a 450/50 nm filter. For both instruments, the geometric mean of the fluorescence across the specified concentrations was calculated by using FlowJo. FSC-A and SSC-A gates were used to isolate yeast populations of interest. Activated cell populations where 60% of the population surpassed the population of the basal fluorescence intensity in the presence of the carrier compound, DMSO, were gated in a subset to isolate cell activation. For plate reader measurements, fluorescence intensity was measured using a Tecan Infinite M1000 Pro. The following excitation, emission, and gain values were used to measure fluorescence intensity (excitation/emission, gain): eGFP (488 nm/507 nm, 221), mKate2 (588 nm/633 nm, 255), mScarlet (569 nm/594 nm, 255), YPet (517 nm/530 nm, 160), and mTq2 (434 nm/474 nm, 221). A bandwidth of 5 nm was used for all excitation and emission wavelengths.

### Data Analysis

To obtain the baseline-corrected data, the geometric mean of sensors in the presence of the carrier solvent DMSO was subtracted from the geometric mean of the sensor in the presence of the compound. Curves were fitted to a nonlinear regression fit (four parameters) were obtained using GraphPad. The operational range was determined by obtaining the EC_10_ and EC_90_ of each curve.

## Supplementary Material



## Notes

Expanded table of hGPCR-based sensors that relied on fluorescent reporters; vector construction; table of materials; table of plasmids; table of strains; table of primers; table of promoters and terminators; GPCR sequences used in this study; colony screen of plasmid-based HTR4-based sensor using five different fluorescent reporters; zoom-ins of the dose–response curves presented in Figure 2A–C; dose–response curve of the double-integrated version of the HTR4-based sensor with mTq2 wt and mTQ2:D134E; sample histograms of the double-integrated HTR4-based sensor with YPet and mTq2 as the reporter using Sony FACS SH800 for signal readout; dose–response of the double-integrated HTR4-based sensor with mScarlet, mTq2 and GFP as the reporter using a fluorescent plate reader (Tecan Infinite M1000 Pro) for signal read out; dose–response curve of the double-integration versions of the HTR1D and Mel1A-based sensors using the P_ADH1_ to drive expression of the hGPCRs; and sequences (PDF)

## Abbreviations

GPCR: G protein-coupled receptor
HTR4: serotonin receptor 4B
MC4R: melanocortin 4 receptor
S1PR2: sphingosine-1-phosphate receptor 2
Mel1A: melatonin receptor 1A
HTR1D: serotonin receptor 1D
HTR1A: serotonin receptor 1A
