# Supplementary material for: Tuning the Response of GPCR-Based Yeast Sensors Using Fluorescent Reporters
Source: ACS Synth Biol. 2025 Dec 15;15(1):61–73. doi: 10.1021/acssynbio.5c00466 (PMC12814776; doi:10.1021/acssynbio.5c00466)
Supplement: Supplementary file 1 [file sb5c00466_si_001.pdf]

Tuning the response of GPCR-based yeast sensors using fluorescent reporters

Ryan Langevin[1#], McKenna Martin-Downey[2#], Amisha Patel[2,3], Haden Archer[1], Sara Davila Severiano[1], Pamela Peralta-Yahya[1,2,3*]

[1] School of Chemistry and Biochemistry, Georgia Institute of Technology, Atlanta, GA 30332

[2] School of Chemical & Biomolecular Engineering, Georgia Institute of Technology, Atlanta, GA 30332
3 Bioengineering Graduate Program, Georgia Institute of Technology, Atlanta, GA 30332

[#] co-first authors

* Email: pperalta-yahya@chemistry.gatech.edu

Supporting Information

Table of contents

Supporting Table 1: Expanded table of GPCR-based sensors that relied on fluorescent reporter S2

Supporting Table 2: Vector construction. S3

Supporting Table 3: Table of Materials S5

Supporting Table 4: Table of Plasmids S6

Supporting Table 5: Table of Strains S7

Supporting Table 6: Table of Primers S9

Supporting Table 7: Table of Promoters and Terminators S11

Supporting Table 8: GPCR sequences used in this study S12

Figure S1. Colony screen of plasmid-based 5-HTR$_{4B}$-based sensor using five different fluorescent reporters. S18

Figure S2. Zoom-ins of the dose response curves presented in Figure 2A-C. S19

Figure SI3. Dose response curve of the double integrated version of the 5-HTR$_{4B}$-based sensor with mTq2 wt and mTq2:D134E. S19

Figure S4. Sample histograms of the double integrated HTR4-based sensor with YPet and mTq2 as the reporter using Sony FACS SH800 for signal readout. S20

Figure S5. Dose response of the double integrated HTR4-based sensor with mScarlet, mTq2 S20
and GFP as the reporter using a fluorescent plate reader (Tecan) for signal read out.

Figure S6. Dose response curve of the double integration versions of the HTR1D and Mel1A-based sensors using the P$_{ADH1}$ to drive expression of the GPCRs. S20

References S21

Supporting Table 1. Expanded table of GPCR-based sensors that relied on fluorescent reporters

Reporter	Human GPCR	Genotype	GPCR promoter	Gα	Ligand	Transcription factor	Reporter promoter	Signal after activation [a]	Chemical exposure [b]	Ref
eGFP	OR1G1	W303 *far1Δ sst2Δ*, ste2Δ, ste12Δ	P_{TEF1}	GPA1	Decanoic acid	Gal4-Ste12	$P_{Gal4(5x)}$	30	4 hrs	[1]
sfGFP	HTR4B	BY4741 *sst2Δ, far1Δ* bar1Δ*, ste2Δ, ste12Δ, gpa1Δ ste3Δ mfα1Δ mfα2Δ mfa1Δ mfa2Δ0 gpr1Δ gpa2Δ* URA3: LexO (6x)-P_{LEU2m}-sfGFP-T_{TDH1}-Spacer 2-P_{PGK1}-GPA1-T_{ENO2}-P_{RAD27}-LexA-PRD-T_{ENO1}-URA3	P_{CCW12}	$G_{\alpha z}$	serotonin	LexA-PRD	LexO(6x)-pLEU2m	63.8	4 hrs	[2]
	A2BR		P_{CCW12}	GPA1	adenosine	pRAD27-LexA-PRD	LexO(6x)-pLEU2m	~325	4 hrs	[3]
	Mel1A		P_{HHF2}	GPA1	melatonin			~150	4 hrs	
	FFAR2	Design 4 *ura3Δ0 ste2Δ0 gpa1Δ0* XI-2:LexO6xLEU2p-HIS3-T_{ENO1}-TEF2P-miRFP670-T_{ADH1}; XII-1:TEF1p-miRFP670-T_{CYC1}; XI-5:P_{PGK1}-GPA1(i1)-T_{ENO2}; XII-2:P_{CCW12}-FFA2R-T_{SSA1}	P_{CCW12}	GPA1/$G\alpha_i$	acetate		LexO6xLEU2p	~11	3 hrs	[4]

Supporting Table 2: Vector construction. All plasmids were sequence verified

DNA sequence	Template	Amplification primers	Landing vector	Landing vector digestion	Final vector	Plasmid name
mKate2	pDONR P4-P1R-mKate2[5]	RP7/RP8	pRS415-Leu2-P_{Fig1}-eGFP[1]	BamHI/HindIII	pRS415-Leu2-P_{Fig1}-mKate2	pRP986
mScarlet	pTriEX-RhoA-wt_mScarlet-i_sGFP2[6]	AM9/AM28		*NcoI/NheI*	pRS415-Leu2-P_{Fig1}-mScarlet	pHW41
YPet	pYPet-His[7]	AM11/AM29			pRS415-Leu2-P_{Fig1}-YPet	pHW42
mTq2: D134E	codon-optimized for *S. cerevisiae*, commercially synthesized	MD25/MD26	pRS415-Leu2-P_{Fig1}-NanoLuc[8]	*NcoI/NheI*	pRS415-Leu2-P_{Fig1}-mTq2:D134E	pMD53
mTq2 WT	pMD53	MD25/MD94 MD26/MD93			pRS415-Leu2-P_{Fig1}-mTq2	pMD100
MC4R	codon-optimized for *S. cerevisiae*, commercially synthesized with overlaps to vector		pESC-HIS3-P_{TEF1}-T_{CYC1} [1]	*BamHI/SacII*	pESC-HIS3-P_{TEF1}-MC4R	pMD4
S1PR2					pESC-HIS3-P_{TEF1}-S1PR2	pMD15
HTR1A					pESC-HIS3-P_{TEF1}-HTR1A	pAP12
Mel1A					pESC-HIS3-P_{TEF1}-Mel1A	pTMC3
P_{TEF1}	pESC-HIS3-P_{TEF1}-5-HTR_{4B}[9]	SD17/SD18	pInt- His3-P_{ADH1}-FLAG-5-HTR_{4B} [10]	*ApaI/XhoI*	pInt-His3-P_{TEF1}-FLAG-HTR4	pSD28
MC4R	pMD4	RL7/RL14	pSD28	*XhoI/SacII*	pInt-His3-P_{TEF1}-MC4R	pRL42
S1PR2	pMD15	RL9/RL14			pInt-His3-P_{TEF1}-S1PR2	pRL43
Mel1A	pTMC3	RL11/RL14			pInt-His3-P_{TEF1}-Mel1A	pRL44
5-HTR_{1D}	pESC-His3-P_{TEF1}-5-HTR_{1D}[9]	RL13/RL14			pInt-His3-P_{TEF1}-HTR1D	pRL53
5-HTR_{1A}	pAP12	AP29/AP30	pNH603-His3-P_{ADH1}-MCP-VP64[11]	*NotI/XhoI*	pInt-His3-P_{ADH1}-HTR1A	pAP130
P_{TEF1}-FLAG-5-HTR_{4B}	pSD28	RL17/RL18	pNH604-Trp1-$P_{TetO(1x)}$- Venus[11]	*ApaI/BamHI*	pInt-Trp1-P_{TEF1}-FLAG-HTR4	pRL51
MC4R	pRL42	RL7/RL14	pRL51	*XhoI/SacII*	pInt-Trp1-P_{TEF1}-MC4R	pRL64
S1PR2	pRL43	RL9/RL14			pInt-Trp1-P_{TEF1}-S1PR2	pRL65
Mel1A	pRL44	RL11/RL14			pInt-Trp1-P_{TEF1}-Mel1A	pRL66
5-HTR_{1D}	pRL53	RL13/RL14			pInt-Trp1-P_{TEF1}-HTR1D	pRL67
5-HTR_{1A}	pAP130	AP83/AP84	pInt-Trp-P_{TEF1}-GPA1_5AA_Gs[10]	*BamHI/XhoI*	pInt-Trp1-P_{TEF1}-HTR1A	pAP277

Mel1A	pRL44	MD111/MD112	pInt-His3-P_{ADH1}-FLAG-5-HTR_{4B}[10]	*XhoI/NotI*	pInt-His3-P_{ADH1}-Mel1A	pRL76
			pInt-Trp1-P_{ADH1}-FLAG-5-HTR_{4B}[10]		pInt-Trp1-P_{ADH1}-Mel1A	pRL77
5-HTR_{1D}	pESC-His3-P_{TEF1}-5-HTR_{1d}[9]	MD114/MD115	pInt-His3-P_{ADH1}-FLAG-5-HTR_{4B}[10]		pInt-His3-P_{ADH1}-HTR1D	pRL78
			pInt-Trp1-P_{ADH1}-FLAG-5-HTR_{4B}[10]		pInt-Trp1-P_{ADH1}-HTR1D	pRL79

Supporting Table 3: Table of Materials

Item	Vendor	Catalog Number
96 Well White Flat Bottom Assay Plate	Costar	3912
96 Well Black/Clear Flat Bottom Assay Plate	Costar	3631
14 mL Polystyrene Round-Bottom Tube	Corning	352057
Flowmi Cell Strainers, 40 µm	SP Bel-Art	136800040
Breathe Easy Sealing Membrane	Electron Microscopy Sciences	70536-10
DMSO	Sigma-Aldrich	34869-100ML
Serotonin Hydrochloride	TCI	S0370
Bremelanotide	Sigma	SML2756
CYM-5520	Sigma	5.31371
Melatonin	Sigma	M5250

Supporting Table 4. Table of Plasmids

**Plasmid Number**	**Plasmid Name**	**Description**	Citation
PPY111	pKM111	pESC-HIS3-P_{TEF1}-tCYC1	[1]
PPY586	pKM586	pRS415-Leu2-P_{FIG1}-eGFP	[1]
PPY889		pDONR P4-P1R-mKate2	[5]
PPY1192	pTMC18	pESC-His3-P_{TEF1}-5-HTR_{4B}	[9]
PPY1421	pAME142	pESC-His3-P_{TEF1}-5-HTR_{1d}	[9]
PPY1670		pYPET-His	[7]
PPY1672		pTriEX-RhoA-wt_mScarlet-i_sGFP2	[6]
PPY1740	pEY15	pRS415-Leu2-pFIG1-NanoLuc	[8]
PPY2162	pJZC530	pNH604-*C. glabrata* Trp1-P_{TetO1x}-Venus-*C. albicans* T_{ADH1}	[11]
PPY2161	pJZC522	pNH603-*C. glabrata* His3-P_{ADH1}-MCP-VP64-*C. albicans* T_{ADH1}	[11]
PPY2735	pPM137	pInt- His3-P_{ADH1}-FLAG-5-HTR_{4B}-	[10]
PPY3481	pPM290	pInt-Trp1-P_{ADH1}-FLAG-5-HTR_{4B}	[10]
PPY2937	pPM189	pInt-Trp1-P_{TEF1}-GPA1_5AA_Gs	[10]
PPY986	pRP986	pRS415-Leu2-P_{FIG1}-mKate2	This work
PPY1760	pHW41	pRS415-Leu2- P_{FIG1}-mScarlet	This work
PPY1761	pHW42	pRS415-Leu2- P_{FIG1}-YPet	This work
PPY2775	pMD53	pRS415-Leu2- P_{FIG1}-mTq2:D134E	This work
PPY3734	pMD100	pRS415-Leu2-P_{FIG1}-mTq2	This work
PPY1190	pTMC3	pESC-His3-P_{TEF1}-Mel1A	This work
PPY2411	pMD4	pESC-His3-P_{TEF1}-MC4R	This work
PPY2426	pMD15	pESC-His3-P_{TEF1}-S1PR2	This work
PPY2451	pAP12	pESC-His3-P_{TEF1}-5-HTR_{1a}	This work
PPY2770	pSD28	pInt-His3-P_{TEF1}-FLAG-HTR4	This work
PPY2929	pRL42	pInt-His3-P_{TEF1}-MC4R	This work
PPY2930	pRL43	pInt-His3-P_{TEF1}-S1PR2	This work
PPY2931	pRL44	pInt-His3-P_{TEF1}-Mel1A	This work
PPY3167	pRL53	pInt-His3-P_{TEF1}-HTR1D	This work

PPY2897	pAP130	pInt-His3-P_{ADH1}-HTR1A	This work
PPY3111	pRL51	pInt-Trp1-P_{TEF1}-FLAG-HTR4	This work
PPY3651	pRL64	pInt-Trp1-P_{TEF1}-MC4R	This work
PPY3652	pRL65	pInt-Trp1-P_{TEF1}-S1PR2	This work
PPY3653	pRL66	pInt-Trp1-P_{TEF1}-Mel1A	This work
PPY3654	pRL67	pInt-Trp1-P_{TEF1}-HTR1D	This work
PPY3519	pAP277	pInt-Trp1-P_{TEF1}-HTR1A	This work
PPY3766	pRL76	pInt-His3-P_{ADH1}-Mel1A	This work
PPY3767	pRL77	pInt-Trp1-P_{ADH1}-Mel1A	This work
PPY3768	pRL78	pInt-His3-P_{ADH1}-HTR1D	This work
PPY3769	pRL79	pInt-Trp1-P_{ADH1}-HTR1D	This work

Supporting Table 5: Table of Strains

**Strain Number**	**Description**	**Citation**
PPY140	*S. cerevisiae* W303 MATa *ade2-1 ura3-1 his3-11 trp1-1 leu2-3 leu2-112 can1-100 Δfar1 Δste2 Δsst2*	[1]
PPY1385	PPY140 transformed with pTMC18 and pKM586	[9]
PPY2772	PPY140 transformed with pTMC18	This work
PPY2474	PPY140 transformed with pTMC18 and pHW42	This work
PPY2475	PPY140 transformed with pTMC18 and pRP986	This work
PPY2476	PPY140 transformed with pTMC18 and pHW41	This work
PPY2773	PPY140 transformed with pTMC18 and pMD53	This work
PPY2480	PPY140 transformed with pMD4	This work
PPY2478	PPY140 transformed with pMD4 and pHW42	This work
PPY2487	PPY140 transformed with pTMC3	This work
PPY2488	PPY140 transformed with pTMC3 and pHW42	This work
PPY2494	PPY140 transformed with pMD15	This work
PPY2495	PPY140 transformed with pMD15 and pHW42	This work
PPY2505	PPY140 transformed with pAME142	This work
PPY2504	PPY140 transformed with pAME142 and pHW42	This work
PPY2785	PPY140 *His3*: P_{TEF1} HTR4	This work

PPY3532	PPY2785 transformed with pKM586	This work
PPY3529	PPY2785 transformed with pRP986	This work
PPY3530	PPY2785 transformed with pHW41	This work
PPY3528	PPY2785 transformed with pHW42	This work
PPY3531	PPY2785 transformed with pMD53	This work
PPY2754	PPY140 *His3*: P_{ADH1}-5-HTR_{4B}	[10]
PPY3844	PPY2754 transformed with pHW42	This work
PPY2955	PPY140 *His3*: P_{TEF1}-MC4R	This work
PPY2972	PPY2955 transformed with pHW42	This work
PPY2956	PPY140 *His3*: P_{TEF1}-S1PR2	This work
PPY2973	PPY2956 transformed with pHW42	This work
PPY2957	PPY140 *His3*: P_{TEF1}-Mel1A	This work
PPY2974	PPY2957 transformed with pHW42	This work
PPY3331	PPY140 *His3*: P_{TEF1}-HTR1D	This work
PPY3370	PPY3331 transformed with pHW42	This work
PPY2997	PPY140 *His3*: P_{ADH1}-HTR1A	This work
PPY3371	PPY2997 transformed with pHW42	This work
PPY3166	PPY140 *His3*: P_{TEF1}-HTR4, *Trp1*: P_{TEF1}-HTR4	This work
PPY3210	PPY3166 transformed with pHW42	This work
PPY3211	PPY3166 transformed with pRP986	This work
PPY3212	PPY3166 transformed with pHW41	This work
PPY3213	PPY3166 transformed with pMD53	This work
PPY3214	PPY3166 transformed with pKM586	This work
PPY3534	PPY140 *His3*: P_{ADH1}-5-$HTR_{4B,}$ Trp1: P_{ADH1}-5-HTR_{4B}	[10]
PPY3739	PPY3534 transformed with HW42	This work
PPY3718	PPY140 *His3*: P_{TEF1}-MC4R, *Trp1*: P_{TEF1}-MC4R	This work
PPY3722	PPY3718 transformed with pHW42	This work
PPY3719	PPY140 *His3*: P_{TEF1}-S1PR2, *Trp1*: P_{TEF1}-S1PR2	This work
PPY3723	PPY3719 transformed with pHW42	This work
PPY3720	PPY140 *His3*: P_{TEF1}-Mel1A, *Trp1*: P_{TEF1}-Mel1A	This work
PPY3724	PPY3720 transformed with pHW42	This work

PPY3721	PPY140 *His3*: P_{TEF1}-HTR1D, *Trp1*: P_{TEF1}-HTR1D	This work
PPY3725	PPY3721 transformed with pHW42	This work
PPY3552	PPY140 *His3*: P_{ADH1}-HTR1A, *TRP1:* P_{TEF1}-HTR1A	This work
PPY3656	PPY3552 transformed with pHW42	This work
PPY3770	PPY140 *TRP1*: P_{ADH1}-Mel1A	This work
PPY3771	PPY140 *TRP1*: P_{ADH1}-Mel1A, *His3*-P_{ADH1}-Mel1A	This work
PPY3765	PPY3771 transformed with pHW42	This work
PPY3772	PPY140 *HIS3*: P_{ADH1}-HTR1D	This work
PPY3773	PPY140 *HIS3*: P_{ADH1}-HTR1D*, Trp1*-P_{ADH1}-HTR1D	This work
PPY3764	PPY3773 transformed with pHW42	This work
PPY3733	PPY3166 transformed with pMD100	This work
PPY3841	PPY140 *Trp1*: P_{TEF1}-HTR4	This work
PPY3842	PPY3841 transformed with pHW42	This work

Supporting Table 6: Table of Primers

Primer Name	Sequence
RP7	ACAAACAAAAAAAAAAAAAAAAAAGGATCC**ATG**GTGAGCGAGCTGATTA
RP8	CGGATCTTAGCTAGCCGCGGTACCAAGCTTTTATCTGTGCCCCAGTTTG
AM9	ACAAACAAAAAAAAAAAAAAAAAAGGATCC**ATG**GTGAGCAAGGGC
AM28	GCGGATCTTAGCTAGCTTACTTGTACAGCTCGTCCATG
AM29	GGTTAGAGCGGATCTTAGCTAGCTTATTTGTACAATTCATTCATACCCTCGG
HW12	CTTTTCGGTTAGAGCGGATC
RL7	TTTAGCTATTTGCTTAGAGCTCCACCGCGGTCAGTATCTGGATGACAAATCGCA
RL9	TTTAGCTATTTGCTTAGAGCTCCACCGCGGTCAGACAACAGTGTTACCTTCCAA
RL11	TTTAGCTATTTGCTTAGAGCTCCACCGCGGTTAAACGGAATCAACCTTGACAAC
RL13	TTTAGCTATTTGCTTAGAGCTCCACCGCGGTTAAGAAGCCTTTCTGAATGGAACAAT
RL14	GCATAGCAATCTAATCTAAGTTTTAATTACAAA
RL17	ATGTGATAACTAATCAGCGGTACCGGGCCCCACACACCATAGCTTCAAAATGT

RL18	ATGTTGCAGGTGTCTAGAACTAGTGGATCCTTAGGTATCAGATGGTTGAGCTG
SD17	GTGGTGGATTCGGCTTTGGGTACCGGGCCCCACACACCATAGCTTCAAAATGT
SD18	GTCGTCGTCGTCTTTGTAGTCCATCTCGAGTTTGTAATTAAAACTTAGATTAGATTGCT**ATG**CTTT
AME535	ATCTAATCTAAGTTTTAATTACAAAGGATCC**ATG**CAAGGTAACGGTTCTG
AME519	TTCGGTTAGAGCGGATCTT
PB140	CCCCCTTTGCTTATAATTGTGTGG
PB141	ACCACCAGAACGGCCGTTAGATC
PB142	AAAAGTTCACCTGTCCCACCTGC
MD111	TCAACTATCTCATATACAATCTCTCTCGAG**ATG**CAAGGTAACGGTTCTG
MD112	TGCTTAGAGCTCCACCGCGGTGGCGGCCGCTTAAACGGAATCAACCTTGACAAC
MD114	TCAACTATCTCATATACAATCTCTCTCGAG**ATG**TCTCCATTGAACCAATCTG
MD115	TGCTTAGAGCTCCACCGCGGTGGCGGCCGCTTAAGAAGCCTTTCTGAATGGAACAA
MD119	TCTAATCTAAGTTTTAATTACAAACTCGAGATGGATGTTCTATCTCCAGGTCAAG
MD120	TGCTTAGAGCTCCACCGC
AP29	TCAACTATCTCATATACAATGTCTCTCGAGATGGATGTTCTATCTCCAGGTCAA
AP30	GCTTAGAGCTCCACCGCGGTGGCGGCCGCTTATTGTCTACAGAACTTACACTTGATGATC
AP83	TCTAATCTAAGTTTTAATTACAAACTCGAGATGGATGTTCTATCTCCAGG
AP84	ATGTTGCAGGTGTCTAGAACTAGTGGATCCTTATTGTCTACAGAACTTACACTTGA
MD93	TCGACTTCAAGGAGGACGGCAACATCCTGGG
MD94	CCAGGATGTTGCCGTCCTCCTTGAAGTCGATG

Start codon **bolded**

Restriction sites used in cloning underlined

Mutation points in red

Supporting Table 7: Table of Promoters and Terminators

Promoter or Terminator	Sequence
P_{TEF1}	CACACACCATAGCTTCAAAATGTTTCTACTCCTTTTTTACTCTTCCAGATTTTC TCGGACTCCGCGCATCGCCGTACCACTTCAAAACACCCAAGCACAGCATAC TAAATTTCCCCTCTTTCTTCCTCTAGGGTGTCGTTAATTACCCGTACTAAAGG TTTGGAAAAGAAAAAAGAGACCGCCTCGTTTCTTTTTCTTCGTCGAAAAAGG CAATAAAAATTTTTATCACGTTTCTTTTTCTTGAAAATTTTTTTTTTTGATTTTTT TCTCTTTCGATGACCTCCCATTGATATTTAAGTTAATAAACGGTCTTCAATTTC TCAAGTTTCAGTTTCATTTTTCTTGTTCTATTACAACTTTTTTTACTTCTTGCTC ATTAGAAAGAAAGCATAGCAATCTAATCTAAGTTTTAATTACAAA
P_{FIG1}	ATCACCCTGCATTGCCTCTTTATTTGACGTTGTTTTTGTAGAACATGAAACGA ATTTTGACTTGATGAGACGAAGTATATATCCAAAGAATACCTTAAATAGAAAA GGAAAGATAATAAATACTAAACACTACTATATATTCAGGTAAAATACAAAAATT ATAACATTTTTAAAACTTTTTTTTTTTGAAAGTCCTTCTCGCTTTAGGATTTTTT CCCATTAAGATTATGATGGTTTCATGTATGTGTCAGTTAAAAAAAATATGGCT AAGTAGCAATGAAACGAACCAAGAAAATGAAAACAATATATAGTGCTGTTGA AATAACAAAGACATTGGTATATATTTGTAAAATGTCTGTTAAATGTTTTTTATC TCAGGTTCTTGCTTGTCTTTGGTAGAAGAAATTATAGTAAACAAACAAACAAA CAAACAAAAAAAAAAAAAAAAA
P_{ADH1}	TAAAACAAGAAGAGGGTTGACTACATCACGATGAGGGGGATCGAAGAAATG ATGGTAAATGAAATAGGAAATCAAGGAGCATGAAGGCAAAAGACAAATATAA GGGTCGAACGAAAAATAAAGTGAAAAGTGTTGATATGATGTATTTGGCTTTG CGGCGCCGAAAAAACGAGTTTACGCAATTGCACAATCATGCTGACTCTGTG GCGGACCCGCGCTCTTGCCGGCCCGGCGATAACGCTGGGCGTGAGGCTGT GCCCGGCGGAGTTTTTTGCGCCTGCATTTTCCAAGGTTTACCCTGCGCTAA GGGGCGAGATTGGAGAAGCAATAAGAATGCCGGTTGGGGTTGCGATGATG ACGACCACGACAACTGGTGTCATTATTTAAGTTGCCGAAAGAACCTGAGTGC ATTTGCAACATGAGTATACTAGAAGAATGAGCCAAGACTTGCGAGACGCGA GTTTGCCGGTGGTGCGAACAATAGAGCGACCATGACCTTGAAGGTGAGACG CGCATAACCGCTAGAGTACTTTGAAGAGGAAACAGCAATAGGGTTGCTACC AGTATAAATAGACAGGTACATACAACACTGGAAATGGTTGTCTGTTTGAGTA CGCTTTCAATTCATTTGGGTGTGCACTTTATTATGTTACAATATGGAAGGGAA CTTTACACTTCTCCTATGCACATATATTAATTAAAGTCCAATGCTAGTAGAGA AGGGGGGTAACACCCCTCCGCGCTCTTTTCCGATTTTTTTCTAAACCGTGGA ATATTTCGGATATCCTTTTGTTGTTTCCGGGTGTACAATATGGACTTCCTCTT TTCTGGCAACCAAACCCATACATCGGGATTCCTATAATACCTTCGTTGGTCT CCCTAACATGTAGGTGGCGGAGGGGAGATATACAATAGAACAGATACCAGA CAGGACATAATGGGCTAAACAAGACTACACCAATTACACTGCCTCATTGATG GTGGTACATAACGAACTAATACTGTAGCCCTAGACTTGATAGCCATCATCAT ATCGAAGTTTCACTACCCTTTTTCCATTTGCCATCTATTGAAGTAATAATAGG CGCATGCAACTTCTTTTCTTTTTTTTTCTTTTCTCTCTCCCCCGTTGTTGTCTC ACCATATCCGCAATGACAAAAAAATGATGGAAGACACTAAAGGAAAAAATTA ACGACAAAGACAGCACCAACAGATGTCGTTGTTCCAGAGCTGATGAGGGGT

	ATCTCGAAGCACACGAAACTTTTTCCTTCCTTCATTCACGCACACTACTCTCT<br>AATGAGCAACGGTATACGGCCTTCCTTCCAGTTACTTGAATTTGAAATAAAAA<br>AAAGTTTGCTGTCTTGCTATCAAGTATAAATAGACCTGCAATTATTAATCTTTT<br>GTTTCCTCGTCATTGTTCTCGTTCCCTTTCTTCCTTGTTTCTTTTTCTGCACAA<br>TATTTCAAGCTATACCAAGCATACAATCAACTATCTCATATACA
T_{ADH1}	TAAGCAAATAGCTAAATTATATACGAATTAATATTATGATTAAGTGTTTACGTG<br>AGTGCGATATTTTTATTACTATCTTATACAGTTGTATATACTCTATAAAATGAG<br>TTGTCTATTAATTAACGCGATGAATGCTTTCTGGGTTTACCTCTCCAACAACT<br>CTAGTTTACTTCTCAATACATTCAATTGTATTTGATTTGTCAATACTTCATCAT<br>TAATCAATTCTATAGTTTTGTTTTTCTCGTTTATTTCCAAATTTAATGCATCAAT<br>TTTATTATTCAATTTGTCGTTGATTTTGGTTAATGATTTTATGGTTTGATCTCT<br>GGCATTGATTGTTTGTGTTAGTTTTTCATTATTGATAATTAAATTATTTAAGTT<br>AGTTATCAACTCGGTGTTTTCAAGTTTCAAGTTTTCAATTTCTTTAGAGTTTAT<br>TAGATTTGTCAAAGTTTCTGAATTGCTTGATTGGTCCTGTAGAAGAGTATTTG<br>TTGTTGTGGATAATTGATTCAATTTTTGAGACAATTGCTGGAAGGCGTTGAAA<br>TATCTAGCATCAATCTCATGGTTTTTTTCCCGAGAGTCTCGTAGATTCAATTG<br>TTTTAATATATCTTGGGACCACTCTTGATTTGAACTCATGGAA
T_{CYC1}	ATCCGCTCTAACCGAAAAGGAAGGAGTTAGACAACCTGAAGTCTAGGTCCC<br>TATTTATTTTTTTATAGTTATGTTAGTATTAAGAACGTTATTTATATTTCAAATT<br>TTTCTTTTTTTTCTGTACAGACGCGTGTACGCATGTAACATTATACTGAAAAC<br>CTTGCTTGAGAAGGTTTTGGGACGCTCGAAG

Supporting Table 8: GPCR Sequences used in this study

Gene	Sequence	Uniprot
HTR4	ATGGATAAGTTGGATGCTAACGTTTCTTCCGAAGAAGGTTTCGGTT<br>CTGTTGAAAAGGTTGTCTTGCTAACATTCTTGTCTACTGTCATCTT<br>GATGGCTATCTTGGGTAACTTGTTGGTTATGGTTGCTGTTTGTTGG<br>GATAGACAATTGAGAAAGATTAAGACTAACTACTTCATTGTTTCCTT<br>GGCTTTCGCTGATTTGTTGGTCTCTGTCTTGGTTATGCCATTCGGT<br>GCTATTGAATTGGTTCAAGATATCTGGATTTACGGTGAAGTCTTCT<br>GTTTGGTTAGAACTTCTTTGGATGTCTTGTTGACTACTGCTTCTATC<br>TTCCATTTGTGTTGTATTTCCTTGGATAGATATTACGCTATCTGTTG<br>TCAACCATTGGTTTACAGAAACAAGATGACTCCATTGAGAATTGCT<br>TTGATGTTGGGTGGTTGTTGGGTCATTCCTACTTTCATCTCTTTCTT<br>GCCAATCATGCAAGGTTGGAACAACATTGGTATCATCGATTTGATT<br>GAAAAGAGAAAGTTCAACCAAAACTCTAACTCTACTTACTGTGTCT<br>TCATGGTTAACAAGCCATACGCTATTACTTGTTCCGTCGTTGCTTT<br>CTACATTCCATTCCTATTGATGGTCTTGGCTTACTACAGAATCTAC<br>GTTACTGCTAAGGAACATGCTCATCAAATCCAAATGTTGCAAAGAG<br>CTGGTGCTTCCTCTGAATCTAGACCACAATCTGCTGATCAACATTC<br>TACTCATAGAATGAGAACTGAAACTAAGGCTGCTAAGACCTTGTGT<br>ATCATCATGGGATGTTTCTGTTTGTGTTGGGCTCCATTCTTCGTTA	Q13639-1

	CTAACATCGTTGATCCATTCATCGATTACACTGTTCCAGGTCAAGTTTGGACTGCTTTCTTGTGGTTGGGTTACATCAACTCTGGTTTGAATCCATTCTTGTACGCATTCTTGAACAAGTCTTTCAGAAGAGCTTTCTTGATCATCTTGTGTTGTGATGATGAAAGATACAGAAGACCATCTATCCTAGGTCAAACTGTTCCTTGTTCTACTACTACTATCAACGGTTCCACACATGTCTTGAGAGATGCTGTTGAATGTGGTGGACAATGGGAATCTCAATGTCATCCACCAGCTACTTCTCCATTGGTTGCAGCTCAACCATCTGATACCTAA	
MC4R	ATGGTTAACTCTACCCATAGAGGTATGCACACTTCATTGCATTTGTGGAACAGATCCTCTTACAGATTGCATTCTAATGCCTCCGAATCTTTAGGTAAGGGTTATTCTGATGGTGGTTGTTACGAACAGTTGTTCGTTTCTCCAGAAGTTTTCGTTACCTTGGGTGTTATTTCCTTGTTGGAAAACATCTTGGTTATCGTTGCTATCGCCAAGAACAAGAACTTGCATTCTCCAATGTACTTTTTCATCTGTTCCTTGGCTGTTGCCGATATGTTGGTTTCTGTTTCTAATGGTTCTGAAACCATCGTCATCACCTTGTTGAATTCTACTGATACCGATGCTCAATCCTTCACCGTTAACATTGATAACGTCATCGACTCCGTTATCTGCTCATCTTTGTTGGCTTCTATTTGCTCCTTGTTGTCCATTGCTGTTGATAGATACTTCACCATTTTCTACGCATTGCAGTACCATAACATCATGACCGTTAAGAGAGTTGGCATTATCATTTCTTGTATTTGGGCTGCTTGTACCGTTTCCGGTATTTTGTTTATTATCTACTCCGATTCCTCCGCCGTTATTATCTGTTTGATTACTATGTTCTTCACCATGTTGGCTTTGATGGCCTCATTATACGTTCACATGTTTTTGATGGCTAGGTTGCACATTAAGAGAATTGCTGTTTTGCCAGGTACTGGTGCTATTAGACAAGGTGCTAATATGAAGGGTGCCATTACTTTGACCATTTTGATCGGTGTTTTCGTTGTTTGTTGGGCTCCATTTTTCTTGCACTTGATCTTCTACATTTCTTGCCCACAAAACCCATACTGTGTTTGTTTCATGTCCCACTTCAACCTGTACCTGATTTTGATTATGTGCAACTCCATCATCGACCCATTGATCTATGCTTTGAGATCCCAAGAATTGCGTAAGACCTTCAAAGAAATTATCTGCTGCTATCCATTAGGTGGTTTGTGCGATTTGTCATCCAGATACTGA	P32245
S1PR2	ATGGGCTCCTTGTATTCTGAATACTTGAACCCAAACAAGGTCCAAGAACATTACAACTACACCAAAGAAACCTTGGAAACTCAAGAAACCACCTCTAGACAAGTTGCTTCTGCTTTCATCGTTATTTTGTGTTGCGCTATCGTTGTCGAAAACTTGTTGGTTTTGATTGCTGTTGCCCGTAACTCTAAGTTTCATTCTGCTATGTACTTGTTCTTGGGTAACTTGGCTGCTTCTGATTTGTTGGCTGGTGTTGCTTTTGTTGCTAACACTTTGTTGTCTGGTTCTGTCACTTTGAGATTGACTCCAGTTCAATGGTTTGCTAGAGAAGGTTCTGCTTTTATTACCTTGTCTGCCTCTGTTTTCTCCTTGTTGGCTATTGCTATTGAAAGACATGTTGCTATCGCCAAGGTTAAGTTATACGGTTCTGATAAGTCTTGCAGGATGTTGTTGTTGATTGGTGCTTCTTGGTTGATCTCCTTGGTTTTAGGTGGTTTGCCAATTTTAGGTTGGAACTGTTTGGGTCATTTGGAAGCTTGTTCTACCGTTTTGCCATTATACGCCAAACATTACGTTTTGTGCGTTGTCACCATTTTCTCCATTA	O95136

	TTTTGTTAGCCATCGTTGCCTTGTACGTCAGAATCTATTGTGTTGTTAGATCCTCTCATGCTGATATGGCTGCTCCACAAACTTTGGCTTTGTTGAAAACTGTTACCATCGTCTTGGGTGTTTTCATTGTCTGTTGGTTGCCAGCTTTCTCTATCTTGTTGTTGGATTATGCTTGCCCAGTTCATTCTTGTCCCATCTTGTACAAAGCTCATTACTTCTTCGCTGTCTCTACCTTGAACTCTTTATTGAACCCAGTTATCTACACCTGGCGTTCTAGAGATTTGAGAAGAGAAGTTCTAAGACCACTACAATGTTGGAGGCCAGGTGTTGGTGTACAAGGTAGAAGAAGAGGTGGTACTCCAGGTCATCATTTGTTGCCTTTGAGATCTTCTTCATCTTTGGAAAGGGGTATGCATATGCCAACTTCTCCAACTTTCTTGGAAGGTAACACTGTTGTCTGA	
Mel1A	ATGCAAGGTAACGGTTCTGCTTTGCCAAACGCTTCCCAACCAGTCTTGAGAGGTGATGGTGCTAGACCATCCTGGTTGGCTTCCGCTTTGGCTTGTGTCTTGATTTTCACTATTGTTGTCGATATTTTGGGTAACTTGTTGGTCATTTTGTCCGTTTACAGAAATAAGAAATTGAGAAACGCTGGTAACATTTTCGTTGTCTCTTTAGCTGTTGCTGATTTGGTCGTTGCTATTTACCCATACCCATTGGTCTTGATGTCCATCTTCAATAACGGTTGGAACTTGGGTTACTTGCATTGTCAAGTTTCCGGTTTCTTGATGGGTTTGTCCGTCATTGGTTCCATTTTCAACATTACCGGTATTGCTATTAACAGATACTGTTACATTTGTCATTCCTTGAAATACGATAAGTTGTATTCTTCCAAGAACTCCTTGTGTTACGTTTTGTTAATTTGGTTGTTGACCTTGGCTGCTGTCTTGCCAAACTTGAGAGCTGGTACCTTGCAATACGATCCTAGAATTTACTCCTGTACCTTCGCTCAATCCGTTTCCTCTGCTTACACTATTGCTGTTGTCGTTTTCCATTTCTTGGTTCCAATGATTATTGTCATTTTCTGTTACTTGAGAATTTGGATTTTGGTCTTGCAAGTCAGACAAAGAGTCAAGCCAGATAGAAAGCCAAAGTTGAAGCCACAAGATTTCAGAAACTTTGTTACCATGTTCGTTGTTTTCGTCTTGTTCGCTATTTGTTGGGCTCCATTGAACTTCATTGGTTTGGCTGTCGCTTCCGATCCAGCTTCCATGGTTCCTAGAATCCCAGAATGGTTGTTCGTTGCTTCTTACTATATGGCTTATTTCAACTCTTGTTTGAACGCTATTATTTACGGTTTGTTGAACCAAAACTTTAGAAAGGAATATAGAAGAATTATTGTTTCCTTATGTACCGCTAGAGTTTTCTTTGTTGATTCCTCTAACGATGTCGCTGATAGAGTTAAGTGGAAACCATCCCCATTGATGACTAACAATAACGTTGTCAAGGTTGATTCCGTTTAA	P48039
HTR1D	ATGTCTCCATTGAACCAATCTGCTGAAGGTTTGCCACAAGAAGCATCCAACAGATCCTTGAACGCTACTGAAACTTCTGAAGCATGGGACCCAAGAACTTTGCAAGCTCTAAAGATCTCCTTGGCTGTTGTCTTGTCAGTCATTACTCTAGCTACTGTCTTGTCTAACGCTTTCGTCTTGACTACTATCTTGTTGACTAGAAAGTTGCATACTCCAGCTAACTACTTGATTGGATCATTGGCTACAACTGATTTGCTAGTTTCCATCTTGGTTATGCCAATCTCCATTGCTTACACTATCACTCATACTTGGAACTTTGGTCAAATCTTGTGTGATATCTGGTTGTCATCTGACATCACTTGTTGTACTGCTTCTATCTTGCATTTGTGTGTCATTGCTTTGGATAGATACTG	P28221

	GGCTATTACTGATGCTTTGGAATACTCTAAGAGAAGAACTGCTGGT<br>CATGCTGCTACTATGATTGCTATTGTCTGGGCTATCTCCATTTGTA<br>TCTCTATTCCACCTTTGTTCTGGAGACAAGCTAAGGCTCAAGAAGA<br>AATGTCTGATTGTTTGGTTAACACTTCTCAAATCTCCTATACTATCT<br>ACTCTACTTGTGGTGCTTTCTACATTCCATCTGTTTTGTTGATCATT<br>CTATACGGTAGAATCTACAGAGCAGCTAGAAACAGAATCTTGAATC<br>CACCATCATTGTATGGTAAGAGATTCACTACTGCTCATTTGATTAC<br>TGGTTCAGCAGGTTCTTCATTGTGTTCCTTGAACTCATCTTTGCAT<br>GAAGGTCATTCTCATTCTGCTGGTTCTCCATTGTTCTTCAATCATG<br>TTAAGATCAAGTTGGCTGATTCTGCATTGGAAAGAAAGAGAATCTC<br>CGCTGCTAGAGAAAGAAAGGCTACTAAGATTCTAGGTATCATTCTA<br>GGTGCTTTCATCATTTGTTGGTTGCCATTCTTTGTCGTTTCCTTGG<br>TCTTGCCAATCTGTAGAGATTCTTGTTGGATTCATCCAGCTTTGTT<br>CGACTTCTTCACTTGGTTGGGATACCTTAACTCTCTTATCAATCCA<br>ATCATCTACACTGTCTTCAACGAAGAATTCAGACAAGCATTCCAAA<br>AGATTGTTCCATTCAGAAAGGCTTCTTAA	
HTR1A	ATGGATGTTCTATCTCCAGGTCAAGGTAACAACACTACTTCTCCAC<br>CAGCTCCATTCGAAACTGGTGGTAACACTACTGGTATCTCTGATGT<br>TACTGTCTCTTATCAAGTCATTACTTCCTTGTTGTTAGGTACATTGA<br>TCTTCTGTGCTGTCTTAGGTAATGCTTGTGTTGTTGCTGCTATTGC<br>ACTAGAAAGATCCTTACAAAACGTTGCTAACTACTTGATTGGATCT<br>TTGGCTGTTACTGATTTGATGGTTTCTGTCTTAGTCTTACCAATGG<br>CTGCTTTGTATCAAGTCTTGAACAAATGGACATTGGGTCAAGTTAC<br>TTGTGATTTGTTCATAGCTTTGGATGTCTTATGTTGTACTTCATCTA<br>TCTTACATTTGTGTGCTATTGCTTTGGATAGATACTGGGCTATCAC<br>TGATCCAATTGATTACGTTAACAAGAGAACTCCAAGAAGAGCAGCT<br>GCATTGATATCATTGACTTGGTTGATTGGTTTCTTGATTTCCATTCC<br>ACCTATGTTAGGTTGGAGAACACCAGAAGATAGATCTGATCCAGA<br>TGCTTGTACTATCTCTAAAGATCATGGTTACACTATCTACTCTACTT<br>TCGGTGCTTTCTACATTCCATTGCTATTGATGTTGGTTCTATACGG<br>TAGAATCTTCAGAGCTGCTAGATTCAGAATCAGAAAGACAGTTAAG<br>AAAGTTGAAAAGACTGGTGCTGATACTAGACATGGTGCTTCTCCA<br>GCTCCACAACCAAAGAAGTCTGTTAACGGTGAATCTGGTTCTAGA<br>AACTGGAGATTAGGTGTTGAATCTAAAGCTGGTGGTGCTTTGTGT<br>GCAAACGGTGCTGTTAGACAAGGTGACGACGGTGCTGCTTTGGAA<br>GTCATTGAAGTTCATAGAGTTGGTAACTCTAAGGAACATTTGCCAT<br>TACCATCTGAAGCTGGTCCAACTCCATGTGCTCCAGCATCATTCG<br>AAAGAAAGAACGAAAGAAACGCTGAAGCTAAGAGAAAGATGGCTT<br>TGGCTAGAGAAAGGAAGACTGTCAAGACTTTGGGTATCATCATGG<br>GAACTTTCATCTTGTGTTGGTTACCTTTCTTCATTGTTGCTTTGGTC<br>CTACCATTCTGTGAATCATCTTGTCATATGCCAACTTTGTTGGGTG<br>CTATCATCAACTGGTTGGGTTACTCTAACTCATTGCTTAACCCAGT	P08908

	TATCTATGCTTACTTCAACAAGGATTTCCAAAACGCTTTCAAGAAG ATCATCAAGTGTAAGTTCTGTAGACAATAA	
YPet	ATGTCTAAAGGTGAAGAATTATTCACTGGTGTTGTCCCAATTTTGG TTGAATTAGATGGTGATGTTAATGGTCACAAATTTTCTGTCTCCGG TGAAGGTGAAGGTGATGCTACGTACGGTAAATTGACCTTAAAATTA CTCTGTACTACTGGTAAATTGCCAGTTCCATGGCCAACCTTAGTCA CTACTTTAGGTTATGGTGTTCAATGTTTTGCTAGATACCCAGATCA TATGAAACAACATGACTTTTTCAAGTCTGCCATGCCAGAAGGTTAT GTTCAAGAAAGAACTATTTTTTTCAAAGATGACGGTAACTACAAGA CCAGAGCTGAAGTCAAGTTTGAAGGTGATACCTTAGTTAATAGAAT CGAATTAAAAGGTATTGATTTTAAAGAAGATGGTAACATTTTAGGT CACAAATTGGAATACAACTATAACTCTCACAATGTTTACATCACTG CTGACAAACAAAAGAATGGTATCAAAGCTAACTTCAAAATTAGACA CAACATTGAAGATGGTGGTGTTCAATTAGCTGACCATTATCAACAA AATACTCCAATTGGTGATGGTCCAGTCTTGTTACCAGACAACCATT ACTTATCCTATCAATCTGCCTTATTCAAAGATCCAAACGAAAAGAG AGACCACATGGTCTTGTTAGAATTTTTGACTGCTGCTGGTATTACC GAGGGTATGAATGAATTGTACAAATAA	
mKate2	ATGGTGAGCGAGCTGATTAAGGAGAACATGCACATGAAGCTGTAC ATGGAGGGCACCGTGAACAACCACCACTTCAAGTGCACATCCGA GGGCGAAGGCAAGCCCTACGAGGGCACCCAGACCATGAGAATCA AGGCGGTCGAGGGCGGCCCTCTCCCCTTCGCCTTCGACATCCTG GCTACCAGCTTCATGTACGGCAGCAAAACCTTCATCAACCACACC CAGGGCATCCCCGACTTCTTTAAGCAGTCCTTCCCCGAGGGCTTC ACATGGGAGAGAGTCACCACATACGAAGACGGGGGCGTGCTGAC CGCTACCCAGGACACCAGCCTCCAGGACGGCTGCCTCATCTACA ACGTCAAGATCAGAGGGGTGAACTTCCCATCCAACGGCCCTGTGA TGCAGAAGAAAACACTCGGCTGGGAGGCCTCCACCGAGACCCTG TACCCCGCTGACGGCGGCCTGGAAGGCAGAGCCGACATGGCCCT GAAGCTCGTGGGCGGGGGCCACCTGATCTGCAACTTGAAGACCA CATACAGATCCAAGAAACCCGCTAAGAACCTCAAGATGCCCGGCG TCTACTATGTGGACAGAAGACTGGAAAGAATCAAGGAGGCCGACA AAGAGACCTACGTCGAGCAGCACGAGGTGGCTGTGGCCAGATAC TGCGACCTCCCTAGCAAACTGGGGCACAGATAA	
mScarlet	ATGGTGAGCAAGGGCGAGGCAGTGATCAAGGAGTTCATGCGGTT CAAGGTGCACATGGAGGGCTCCATGAACGGCCACGAGTTCGAGA TCGAGGGCGAGGGCGAGGGCCGCCCCTACGAGGGCACCCAGAC CGCCAAGCTGAAGGTGACCAAGGGTGGCCCCCTGCCCTTCTCCT GGGACATCCTGTCCCCTCAGTTCATGTACGGCTCCAGGGCCTTCA TCAAGCACCCCGCCGACATCCCCGACTACTATAAGCAGTCCTTCC CCGAGGGCTTCAAGTGGGAGCGCGTGATGAACTTCGAGGACGGC GGCGCCGTGACCGTGACCCAGGACACCTCCCTGGAGGACGGCA CCCTGATCTACAAGGTGAAGCTCCGCGGCACCAACTTCCCTCCTG	

	ACGGCCCCGTAATGCAGAAGAAGACAATGGGCTGGGAAGCGTCC ACCGAGCGGTTGTACCCCGAGGACGGCGTGCTGAAGGGCGACAT TAAGATGGCCCTGCGCCTGAAGGACGGCGGCCGCTACCTGGCGG ACTTCAAGACCACCTACAAGGCCAAGAAGCCCGTGCAGATGCCC GGCGCCTACAACGTCGACCGCAAGTTGGACATCACCTCCCACAA CGAGGACTACACCGTGGTGGAACAGTACGAACGCTCCGAGGGCC GCCACTCCACCGGCGGCATGGACGAGCTGTACAAGTAA	
mTq2	ATGGTGAGCAAGGGCGAGGAGCTGTTCACCGGGGTGGTGCCCAT CCTGGTCGAGCTGGACGGCGACGTAAACGGCCACAAGTTCAGCG TGTCCGGCGAGGGCGAGGGCGATGCCACCTACGGCAAGCTGAC CCTGAAGTTCATCTGCACCACCGGCAAGCTGCCCGTGCCCTGGC CCACCCTCGTGACCACCCTGTCCTGGGGCGTGCAGTGCTTCGCC CGCTACCCCGACCACATGAAGCAGCACGACTTCTTCAAGTCCGCC ATGCCCGAAGGCTACGTCCAGGAGCGCACCATCTTCTTCAAGGAC GACGGCAACTACAAGACCCGCGCCGAGGTGAAGTTCGAGGGCGA CACCCTGGTGAACCGCATCGAGCTGAAGGGCATCGACTTCAAGG AGGACGGCAACATCCTGGGGCACAAGCTGGAGTACAACTACTTTA GCGACAACGTCTATATCACCGCCGACAAGCAGAAGAACGGCATCA AGGCCAACTTCAAGATCCGCCACAACATCGAGGACGGCGGCGTG CAGCTCGCCGACCACTACCAGCAGAACACCCCCATCGGCGACGG CCCCGTGCTGCTGCCCGACAACCACTACCTGAGCACCCAGTCCA AGCTGAGCAAAGACCCCAACGAGAAGCGCGATCACATGGTCCTG CTGGAGTTCGTGACCGCCGCCGGGATCACTCTCGGCATGGACGA GCTGTACAAGTAA	
eGFP	ATGGTGAGCAAGGGCGAGGAGCTGTTCACCGGGGTGGTGCCCAT CCTGGTCGAGCTGGACGGCGACGTAAACGGCCACAAGTTCAGCG TGTCCGGCGAGGGCGAGGGCGATGCCACCTACGGCAAGCTGAC CCTGAAGTTCATCTGCACCACCGGCAAGCTGCCCGTGCCCTGGC CCACCCTCGTGACCACCCTGACCTACGGCGTGCAGTGCTTCAGC CGCTACCCCGACCACATGAAGCAGCACGACTTCTTCAAGTCCGCC ATGCCCGAAGGCTACGTCCAGGAGCGCACCATCTTCTTCAAGGAC GACGGCAACTACAAGACCCGCGCCGAGGTGAAGTTCGAGGGCGA CACCCTGGTGAACCGCATCGAGCTGAAGGGCATCGACTTCAAGG AGGACGGCAACATCCTGGGGCACAAGCTGGAGTACAACTACAAC AGCCACAACGTCTATATCATGGCCGACAAGCAGAAGAACGGCATC AAGGTGAACTTCAAGATCCGCCACAACATCGAGGACGGCAGCGT GCAGCTCGCCGACCACTACCAGCAGAACACCCCCATCGGCGACG GCCCCGTGCTGCTGCCCGACAACCACTACCTGAGCACCCAGTCC GCCCTGAGCAAAGACCCCAACGAGAAGCGCGATCACATGGTCCT GCTGGAGTTCGTGACCGCCGCCGGGATCACTCTCGGCATGGACG AGCTGTACAAGTAA	N/A

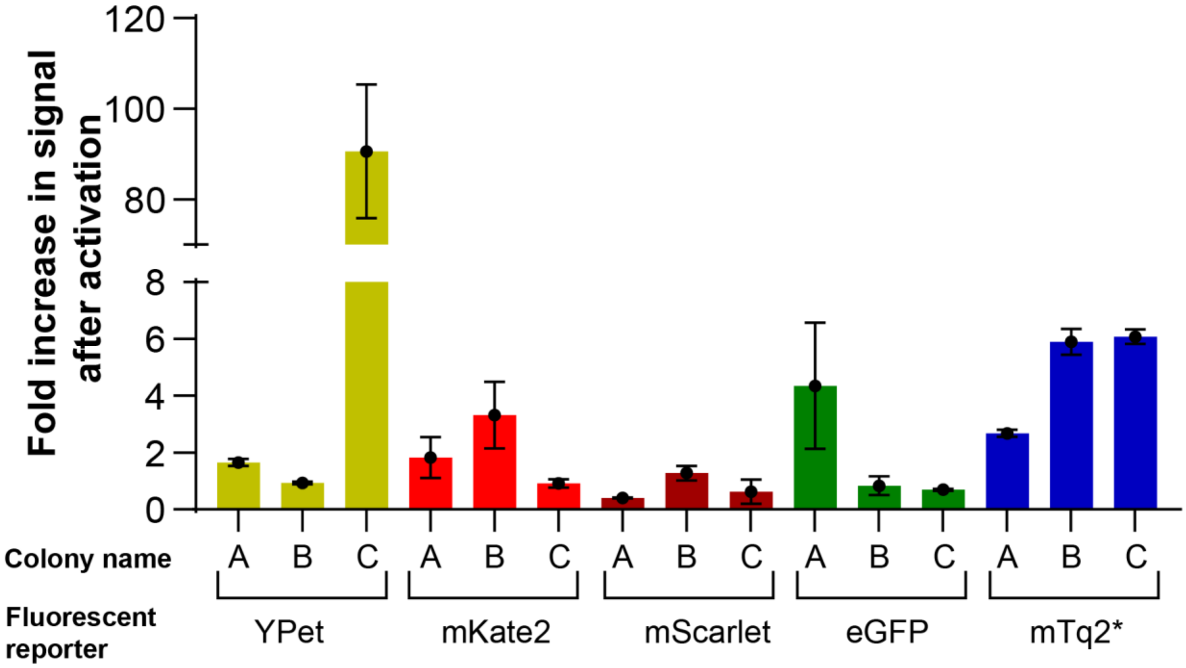

Figure S1. Colony screen of plasmid-based 5-HTR$_{4B}$-based sensor using five different fluorescent reporters. The fold increase in signal after activation was determined by dividing the signal obtained in the presence of 10^{-4} M serotonin over the signal obtained in the presence of the carrier solvent DMSO. Shown are the mean and standard error of the mean of technical replicates (n=3) of three independent colonies (A-C) for each fluorescent reporter analyzed.

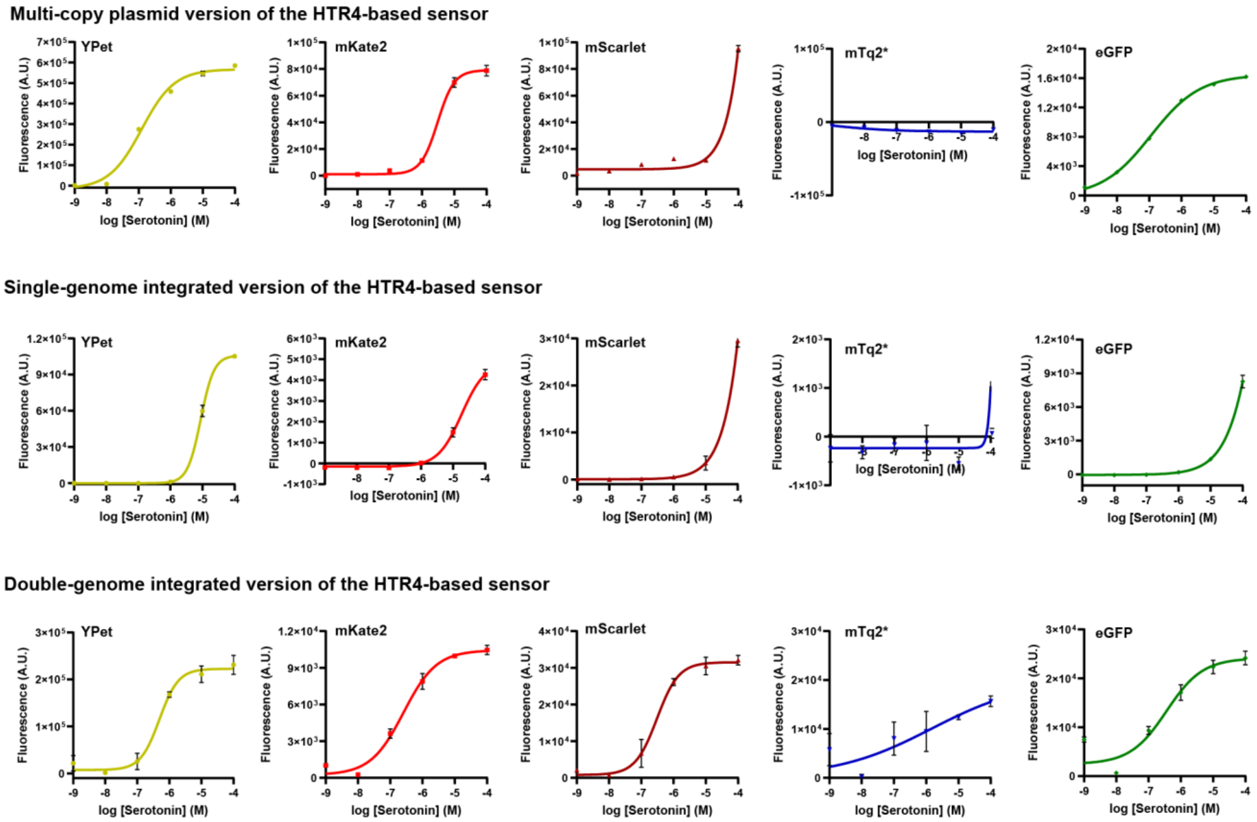

Figure S2. Zoom-ins of the dose response curves presented in Figure 2A-C.

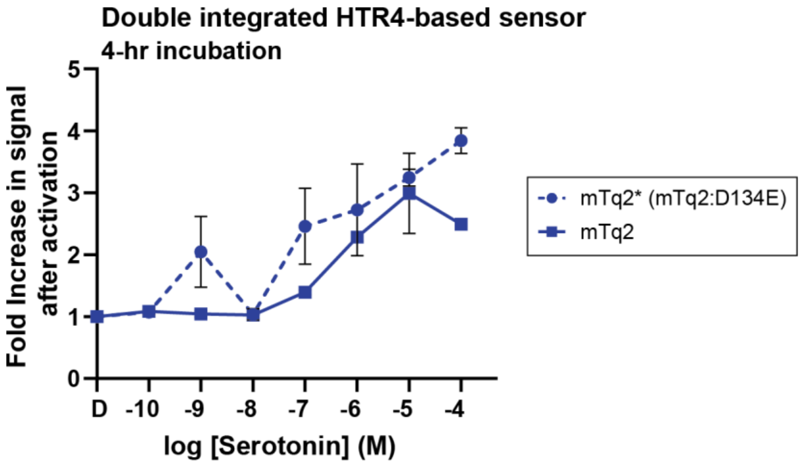

Figure S3. Dose response curve of the double integrated version of the 5-HTR$_{4B}$-based yeast sensor with mTq2 wt and mTq2:D134E. Shown are the mean and standard error of the mean (SEM), biological replicates, n=3.

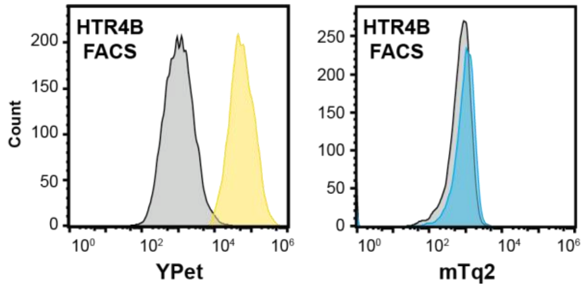

Figure S4. Sample histograms of the double integrated HTR4-based sensor with YPet and mTq2 as the reporter using Sony FACS SH800 for signal readout.

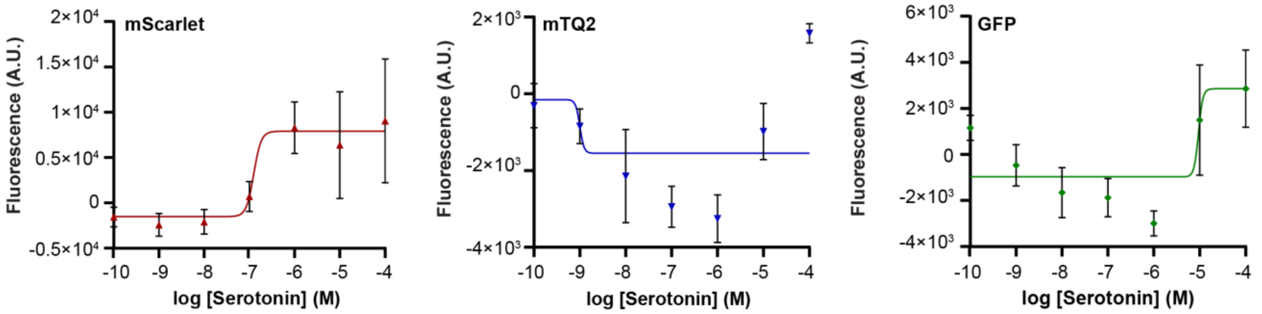

Figure S5. Dose response of the double integrated HTR4-based sensor with mScarlet, mTq2 and GFP as the reporter using a fluorescent plate reader (Tecan) for signal read out.

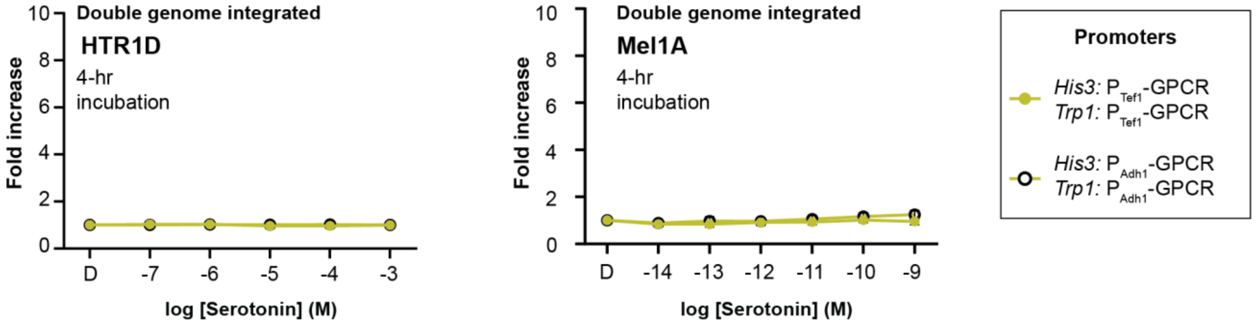

Figure S6. Dose response curve of the double integration versions of the 5-HTR$_{1D}$- and Mel1A-based sensors using the P$_{ADH1}$ to drive expression of the GPCRs. Shown are the mean and standard error of the mean (SEM), biological replicates, n=3.

References

1. Mukherjee, K.; Bhattacharyya, S.; Peralta-Yahya, P. GPCR-Based Chemical Biosensors for Medium-Chain Fatty Acids. *ACS Synth Biol* **2015**, *4* (12), 1261-1269. DOI: 10.1021/sb500365m.
2. Lengger, B.; Hoch-Schneider, E. E.; Jensen, C. N.; Jakociu Nas, T.; Petersen, A. A.; Frimurer, T. M.; Jensen, E. D.; Jensen, M. K. Serotonin G Protein-Coupled Receptor-Based Biosensing Modalities in Yeast. *ACS Sens* **2022**, *7* (5), 1323-1335. DOI: 10.1021/acssensors.1c02061.
3. Shaw, W. M.; Yamauchi, H.; Mead, J.; Gowers, G. O. F.; Bell, D. J.; Oling, D.; Larsson, N.; Wigglesworth, M.; Ladds, G.; Ellis, T. Engineering a Model Cell for Rational Tuning of GPCR Signaling. *Cell* **2019**, *177* (3), 782-+. DOI: 10.1016/j.cell.2019.02.023.
4. Clausen Lind, A.; De Castro Gomes, D.; Bisquert, R.; Mårtensson, J.; Sundqvist, M.; Forsman, H.; Dahlgren, C.; David, F.; Siewers, V. Development of a yeast-based sensor platform for evaluation of ligands recognized by the human free fatty acid 2 receptor. *FEMS Yeast Res* **2025**, *25* DOI: 10.1093/femsyr/foaf001
5. Buj, R.; Iglesias, N.; Planas, A. M.; Santalucia, T., A plasmid toolkit for cloning chimeric cDNAs encoding customized fusion proteins into any Gateway destination expression vector. *BMC Mol Biol* **2013**, *14* (1), 18. DOI: 10.1186/1471-2199-14-18
6. Bindels, D. S.; Haarbosch, L.; van Weeren, L.; Postma, M.; Wiese, K. E.; Mastop, M.; Aumonier, S.; Gotthard, G.; Royant, A.; Hink, M. A.; et al. mScarlet: a bright monomeric red fluorescent protein for cellular imaging. *Nat Methods* **2017**, *14* (1), 53-56. DOI: 10.1038/nmeth.4074.
7. Nguyen, A. W.; Daugherty, P. S. Evolutionary optimization of fluorescent proteins for intracellular FRET. *Nat Biotechnol* **2005**, *23* (3), 355-360. DOI: 10.1038/nbt1066.
8. Yasi, E. A.; Allen, A. A.; Sugianto, W.; Peralta-Yahya, P. Identification of Three Antimicrobials Activating Serotonin Receptor 4 in Colon Cells. *ACS Synth Biol* **2019**, *8* (12), 2710-2717. DOI: 10.1021/acssynbio.9b00310.
9. Ehrenworth, A. M.; Claiborne, T.; Peralta-Yahya, P. Medium-Throughput Screen of Microbially Produced Serotonin via a G-Protein-Coupled Receptor-Based Sensor. *Biochemistry* **2017**, *56* (41), 5471-5475. DOI: 10.1021/acs.biochem.7b00605.
10. Marquez-Gomez, P. L.; Damiano, S. R.; Torp, L. R.; Peralta-Yahya, P. Modulating the Properties of GPCR-Based Sensors Via C-Terminus Isoforms. *ACS Synth Biol* **2025**. *14* (5), 1853-1860 DOI: 10.1021/acssynbio.4c00847.
11. Zalatan, J. G.; Lee, M. E.; Almeida, R.; Gilbert, L. A.; Whitehead, E. H.; La Russa, M.; Tsai, J. C.; Weissman, J. S.; Dueber, J. E.; Qi, L. S. Engineering complex synthetic transcriptional programs with CRISPR RNA scaffolds. *Cell* **2015**, *160* (1), 339-350. DOI: 10.1016/j.cell.2014.11.052
